# Associations Between Symptoms of Depression and Air Pollutant Exposure Among Older Adults: Results From the Taiwan Longitudinal Study on Aging (TLSA)

**DOI:** 10.3389/fpubh.2021.779192

**Published:** 2022-01-12

**Authors:** Kuan-Chin Wang, Yuan-Ting C. Lo, Chun-Cheng Liao, Yann-Yuh Jou, Han-Bin Huang

**Affiliations:** ^1^School of Public Health, National Defense Medical Center, Taipei, Taiwan; ^2^Department of Family Medicine, Taichung Armed Forces General Hospital, Taichung, Taiwan; ^3^Health Promotion Administration, Ministry of Health and Welfare, Taipei, Taiwan

**Keywords:** depressive symptoms, older adults, air pollution, repeated-measures study, cohort study

## Abstract

**Background:** Little epidemiological research has investigated the associations of air pollutant exposure over various time windows with older adults' symptoms of depression. This study aimed to analyze the relationships of long- and short-term ambient air pollution exposure (to coarse particulate matter, O_3_, SO_2_, CO, and NO_x_) with depressive symptoms in a sample of community-dwelling older adults.

**Methods:** A sample of older adults (*n* = 1,956) was recruited from a nationally representative multiple-wave study (Taiwan Longitudinal Study on Aging). Between 1996 and 2007, four waves of surveys investigated depressive symptoms by using the 10-item Center for Epidemiologic Studies Depression questionnaire. We approximated air pollutant concentrations from 1995 to 2007 by using daily concentration data for five air pollutants at air quality monitoring stations in the administrative zone of participants' residences. after adjusting for covariates, we applied generalized linear mixed models to analyze associations for different exposure windows (7-, 14-, 21-, 30-, 60-, 90-, and 180-day and 1-year moving averages).

**Results:** In a one-pollutant model, long- and short-term exposure to CO and NO_x_ was associated with heightened risks of depressive symptoms; the odds ratio and corresponding 95% confidence interval for each interquartile range (IQR) increment in CO at 7-, 14-, 21-, 30-, 60-, 90-, and 180-day and 1-year moving averages were 1.232 (1.116, 1.361), 1.237 (1.136, 1.348), 1.216 (1.128, 1.311), 1.231 (1.133, 1.338), 1.224 (1.124, 1.332), 1.192 (1.106, 1.285), 1.228 (1.122, 1.344), and 1.180 (1.102, 1.265), respectively. Those for each IQR increment in NO_x_ were 1.312 (1.158, 1.488), 1.274 (1.162, 1.398), 1.295 (1.178, 1.432), 1.310 (1.186, 1.447), 1.345 (1.209, 1.496), 1.348 (1.210, 1.501), 1.324 (1.192, 1.471), and 1.219 (1.130, 1.314), respectively. The exposure to PM_10_, O_3_, and SO_2_ over various windows were not significant. In the two-pollutant model, only the associations of NO_x_ exposure with depressive symptoms remained robust after adjustment for any other pollutant.

**Conclusions:** Exposure to traffic-associated air pollutants could increase depression risks among older adults.

## Introduction

Depression, a common mental health issue, is among the key causes of disability. Worldwide, approximately 264 million individuals have depression (1). Among all nonfatal diseases worldwide, depressive disorder has the third highest burden (1). Negative health effects associated with depression include poor social function, reduced quality of life, and heightened cancer mortality risks (2, 3). Depression is a major public health challenge worldwide, and risk factor management is crucial for its prevention.

Air pollution, both ambient and indoor, considerably affects environments worldwide. According to one report, in 2017, air pollution caused 4.90 million deaths and was associated with the loss of 1.47 billion disability-adjusted life years (4). The deleterious effect that air pollution exerts on depression and various other mental health disorders has recently gained attention worldwide, especially in public health-care research (5–7).

Depression is among the commonest mental health disorders affecting the older adult population (8). Epidemiological investigations have examined associations of air pollution exposure with depression in older adult populations (9–16), but results have been limited and inconsistent. Whether air pollution over different exposure windows (e.g., short-term vs. long-term) is linked to depression remains unclear, as does whether specific air pollutants are related to depression in older adults. Furthermore, epidemiological research regarding the joint effects of cognitive function and air pollution over various exposure windows on depression in the older population is scarce. To make up for these deficiencies, our aims were to explore the relationship between air pollution over various exposure windows and depressive symptoms by conducting repeated measurements in Taiwanese older adults; additionally, we examined the joint effects of long- or short-term exposure to air pollution and cognitive decline on depressive symptoms.

## Methods

### Participants

The Taiwan Longitudinal Study on Aging (TLSA), a survey with national representativeness, was initiated in 1989 by the Health Promotion Administration of Taiwan's Ministry of Health and Welfare (17). In the TLSA, equal-probability sampling was implemented in three stages to select 4,049 older adults (≥60 years); in the initial survey year, a 92% response rate was achieved (17). Follow-up surveys were conducted in 1993, 1996, 1999, 2003, and 2007. Individuals who participated in the survey provided only verbal consent before 2007; however, in 2007, signed consent forms were provided by each participant prior to enrolment by the Health Promotion Administration.

**Figure 1:**
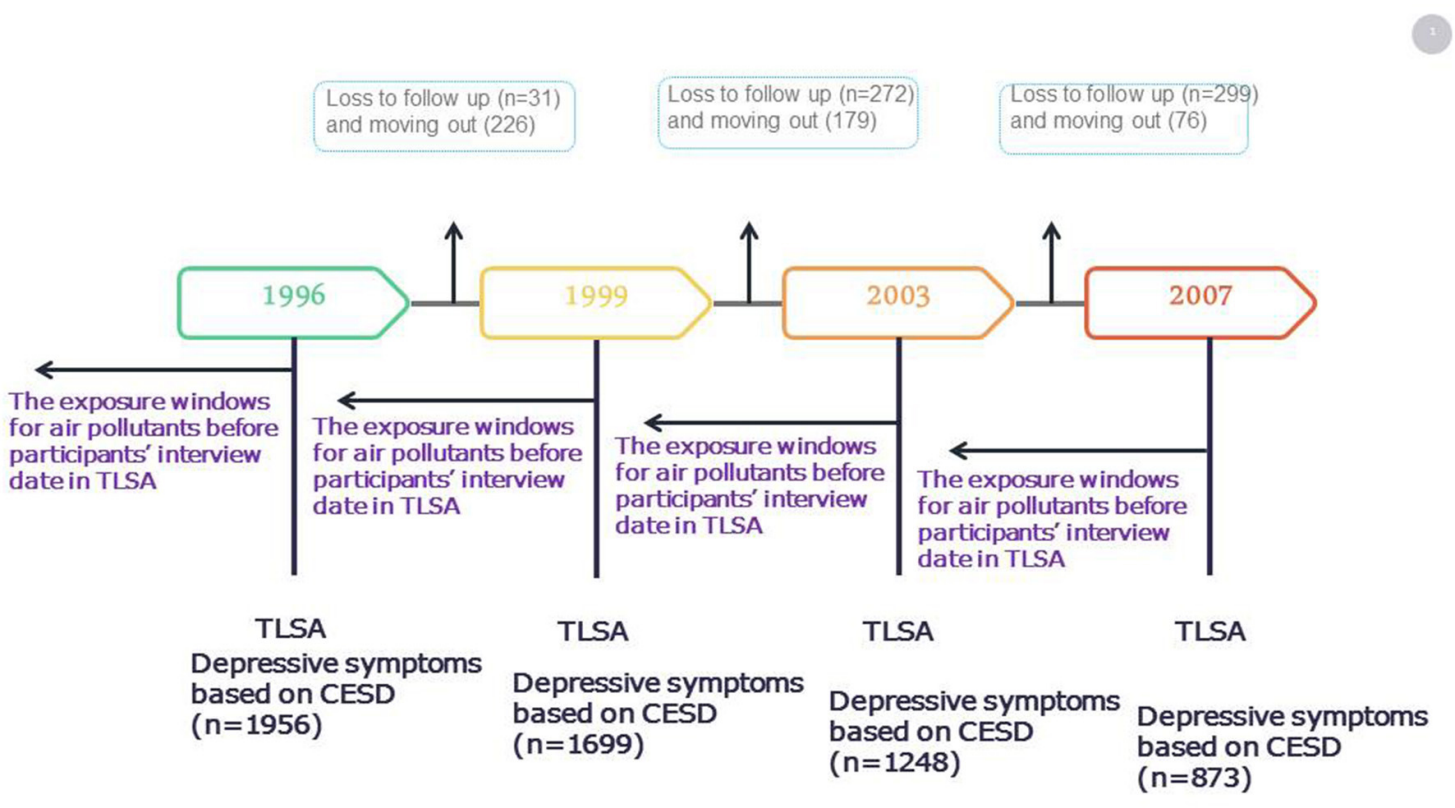
Sample selection flowchart.

In the current study, data for the years 1996–2007 were used. In total, 1,956 older adults (≥65 years) were included in the 1996 survey after the exclusion of participants with a stroke history in 1993, those with a Short Portable Mental Status Questionnaire (SPMSQ) score of <3 in 1993, those with incomplete information, and those who had moved residence during the study period (Figure 1). A total of 1,956 participants were included in 1996. During *1996–1999*, there were 31 participants lost to follow-up, and 226 participants moved during follow-up. Follow by *2003, 2*72 participants were lost to follow-up, and 179 participants moved during follow-up. Till *2007, 2*99 participants were lost to follow-up, and 76 participants moved during follow-up. During 1996 to 2007, total of 5776 number of observations were presented in the present study. Each participant had about 3 follow-up times. The percentage of loss to follow-up and moving out was 7.7 and 6.1% in this study, respectively.

Approval for this study was granted by the Tri-Service General Hospital Institutional Review Board (No.: 2-104-05-153).

### Depressive Symptom Measurement

Symptoms of depression were assessed with the 10-item version of the Center for Epidemiological Studies Depression (CES-D-10) questionnaire. The CES-D-10 achieves similar precision to that of the 20-item CES-D (18). The Cronbach α for internal consistency is 0.78–0.87 for the TLSA sample (19). The CES-D-10 requires participants to respond to *ten* items (see Supplementary Table S1) and indicate how often in the preceding week they experienced the feeling described, from 0 (rarely or never) to 3 (most of or almost all of the time). The score range is 0–30; more severe symptoms of depression are indicated by higher scores. Following Chang and Weng (20), we considered participants with a CES-D-10 score of ≥10 to have moderate-to-severe depressive symptoms.

### Exposure Assessment

Hourly data on particulate matter (PM) with a diameter of <10 μm (PM_10_), O_3_, CO, SO_2_, NO, NO_2_, and NOx concentrations were obtained from monitoring stations (*N* = 75) established by Taiwan's Environmental Protection Administration (TEPA) (21), all of which are located on the main island of Taiwan (see Supplementary Figure S1); these 75 stations provided continual measurements from 1995 to 2007. Stringent and independent quality assurance and control procedures were implemented to ensure the quality of the meteorological data: with TEPA authorization, a private company conducted yearly audits and routine inspection of the performance of the monitoring instruments. Average daily measurements of seven air pollutants at each station were employed in later analyses. For a day to be included in calculation, ≥75% of the hourly values must be available. The pollutant concentrations measured at monitoring stations within the city or county of a TLSA participant were assigned to that participant, as described elsewhere (22). Along with 1-year moving averages, 7-, 14-, 21-, 30-, 60-, 90-, and 180-day moving averages prior to each participant's survey date were calculated.

### Covariates

Data pertaining to related covariates were gathered using structured interviews. Demographics (e.g., educational attainment, age, marital and economic status) and information on the participants' lifestyle (e.g., physical activity, smoking status, and alcohol intake) were obtained. The instrumental activities of daily living (IADLs) score was determined to reflect the participants' physical function. IADLs include telephone use, financial management, independent public transport use, strenuous housework, and light housework. The overall score range is 0–18, with low physical function denoted by a higher score. We examined cognitive function using a 5-item SPMSQ (SPMSQ-5), which was validated using a version of the Mini-Mental State Examination in Chinese (23). The SPMSQ-5 was applied to measure cognitive function in the Asset and Health Dynamics Among the Oldest Old study (24, 25). Correct answers are worth 1 point, with the overall scores ranging from 0 to 5. An SPMSQ-5 score of <3 indicates moderate-to-severe cognitive impairment (26). In this study, participants self-reported chronic diseases, such has heart disease, diabetes, and hypertension, and these were verified with a physician's diagnosis.

### Statistical Analysis

Because repeated measures and a prospective design were used, we employed a generalized linear mixed model–based procedure (PROC GLIMMIX) to examine the relationship of depression symptoms with exposure to ambient PM_10_, O_3_, CO, SO_2_, NO, NO_2_, and NOx, while accounting for the random effects of repeated measurements. We modeled moderate-to-severe depressive symptoms as a binary outcome indicated by a CES-D-10 score of ≥10. We investigated the PM_10_, O_3_, CO, SO_2_, NO, NO_2_, and NOx exposure by using window averages from 7 days up to 1 year before each interview date to evaluate the effect of long- and short-term air pollutant exposure on depressive symptoms. To address non-linear relationships, we categorized the exposures into quartiles to examine the associations. Furthermore, we applied the residual regression models due to the high correlation between different air pollutants in two-pollutant models (27).

Variables related to outcomes or exposure, rather than intermediate variables between exposure and outcomes were selected as confounders (28). Additional confounders were selected on the basis of a 10% change-in-estimate criterion (29); adjusted confounders were sex, age, marital situation, financial situation, educational attainment, alcohol intake, physical activity level, heart disease status, IADL score, season of study visit, and SPMSQ-5 score. To investigate the joint effect of cognitive function with pollutant exposure on depressive symptoms, air pollutant–cognitive function interaction terms were included in these models. We performed all analyses adjusted confounders included by using SAS 9.3 (SAS Institute Inc., Cary, NC, USA) with a significance threshold of *p* < 0.05.

## Results

### Demographics

The demographics of the participants surveyed from 1996 to 2007 are presented in Table 1. The mean participant age was 73.4 years at baseline, and approximately 60% of the participants were men. More than 63% of the participants had a spouse. Approximately half of the participants had a primary and secondary degree, and 43% self-evaluated their financial status as fair. Large proportions of the participants engaged in physical activity on a regular basis, did not consume alcohol, did not smoke, and did not have hypertension or diabetes mellitus. The diabetes mellitus and hypertension rates in 2007 were above those in 1996. There were 4.90% of the study group had an SPMSQ score of <3 and 1.81 was the mean IADL score in 1996. There were 18.1% of participants had an SPMSQ score of <3 and 4.13 was the mean IADL score in 2007.

**Table 1:** TLSA participant data for each study year.

**Variable**	**Year 1996 (*n =* 1,956)**	**Year 1999 (*n =* 1,699)**	**Year 2003 (*n =* 1,248)**	**Year 2007 (*n =* 873)**
Male, *n* (%)	1172 (59.9)	997 (58.7)	727 (58.3)	462 (52.9)
Age, y, mean ± SD	73.44 ± 4.90	76.10 ± 4.68	78.64 ± 4.04	82.28 ± 3.75
Spouse, yes	1,233 (63.0)	1,006 (59.2)	673 (53.9)	403 (46.2)
**Personal education**, ***n*** **(%)**				
Illiterate	664 (33.9)	555 (32.6)	379 (30.4)	284 (32.5)
Primary and secondary school	1,047 (53.5)	927 (54.6)	689 (55.2)	459 (52.6)
High school and above	245 (12.5)	217 (12.8)	180 (14.4)	130 (14.8)
**Self-reported financial status**, ***n*** **(%)**				
Very satisfied	163 (8.30)	125 (7.40)	71 (5.70)	47 (5.38)
Satisfied	655 (33.5)	561 (33.0)	515 (41.3)	365 (41.7)
Fair	843 (43.1)	678 (39.9)	427 (34.2)	309 (35.4)
Dissatisfied	243 (12.4)	250 (14.7)	180 (14.4)	115 (13.2)
Very dissatisfied	52 (2.70)	85 (5.00)	55 (4.40)	37 (4.24)
Physical activity, *n* (%)	1,164 (59.5)	1,099 (64.7)	844 (67.6)	589 (67.5)
Smoking status	542 (27.7)	400 (23.5)	231 (18.5)	107 (12.3)
Alcohol-drinking	381 (19.5)	380 (22.4)	265 (21.2)	170 (19.5)
**Season of study visit**				
Spring	1,190 (60.8)	1,493 (87.9)	–	352 (40.3)
Summer	714 (36.5)	173 (10.2)	–	511 (58.5)
Fall	52 (2.7)	31 (1.8)	1,142 (91.5)	10 (1.15)
Winter	–	2 (0.1)	106 (8.5)	–
Hypertension	512 (26.2)	633 (37.5)	540 (43.3)	406 (46.5)
Diabetes	192 (9.80)	254 (14.9)	203 (16.3)	121 (13.9)
Heart disease	308 (15.7)	393 (23.1)	332 (26.6)	223 (25.5)
SPMSQ (0–5)				
≥3	1,861 (95.1)	1606 (94.5)	1,155 (92.5)	715 (81.9)
<3	95 (4.90)	93 (5.50)	93 (7.50)	158 (18.1)
IADL (0–18), mean ± SD	1.81 ± 3.48	2.27 ± 3.91	3.10 ± 4.42	4.13 ± 5.03

*TLSA, taiwan longitudinal study on aging; SPMSQ, short portable mental status questionnaire; IADL, instrumental activities of daily living*.

### Distribution of Depressive Symptoms and Exposure to Air Pollutants for Different Exposure Windows by Survey Year

Participants with a CES-D-10 score of ≥10 were around 20.5% to 25.8% in four survey years. The concentrations of air pollutants for different exposure windows by survey year. We discovered that the PM_10_, CO, SO_2_, NO, NO_2_, and NOx levels in the 1-year and 7-day exposure windows declined from 1995 to 2007 (*p* for trend: < 0.001) (Table 2). The levels of air pollutants (PM_10_, CO, SO_2_, NO, NO_2_, and NOx) for other exposure windows (14-, 21-, 30-, 60-, 90-, and 180-day) were also declined from 1995 to 2007 (Supplementary Table S2). However, the O3 levels for the 1-year and 21-, 30-, 60-, 90-, and 180-day exposure windows gradually increased from 1996 to 2007 (*p* for trend: < 0.001). During the study period, the levels of O3 for the 7- and 14-day exposure windows declined (*p* for trend: < 0.001). The percentile 25th, 50th, 75th, and interquartile range (IQR) were presented in Supplementary Table S3. We observed that the correlations between short-and long-term averages for the same pollutant were ranged from 0.7 to 0.9 except O_3_ (Supplementary Table S4). We also found that the correlations between CO and NOx for the different exposure window were ranged from 0.8 to 0.9 and the other pollutants presented the low to moderate correlations (Supplementary Table S5).

**Table 2:** Distribution of depressive score and air pollutant concentrations over various exposure windows for each survey year.

**Variable**	**Year 1996 (*n* = 1,956)**	**Year 1999 (*n* = 1,699)**	**Year 2003 (*n* = 1,248)**	**Year 2007 (*n* = 873)**	***P* for trend**
	**Mean ± SD**	**Mean ± SD**	**Mean ± SD**	**Mean ± SD**	
**CES-D (0–30)**					
<10, n (%)	1468 (75.1)	1277 (75.2)	992 (79.5)	650 (74.2)	–
≥10, n (%)	488 (24.9)	422 (24.8)	256 (20.5)	226 (25.8)	–
**7-day**					
PM_10_ (μg/m^3^)	61.50 ± 15.47	69.95 ± 25.32	53.79 ± 19.94	48.62 ± 20.86	<0.001
O_3_ (ppb)	34.05 ± 4.05	32.38 ± 5.72	31.61 ± 7.68	29.99 ± 10.62	<0.001
CO (ppm)	0.757 ± 0.242	0.687 ± 0.180	0.643 ± 0.141	0.474 ± 0.177	<0.001
SO_2_ (ppb)	6.40 ± 3.92	4.99 ± 2.90	2.99 ± 1.64	4.01 ± 1.31	<0.001
NO (ppb)	9.76 ± 8.45	8.37 ± 6.87	6.33 ± 5.12	6.35 ± 5.19	<0.001
NO_2_ (ppb)	24.18 ± 6.12	24.73 ± 5.47	19.84 ± 5.67	15.72 ± 6.86	<0.001
NOx (ppb)	33.93 ± 12.84	32.96 ± 10.89	26.13 ± 9.43	22.08 ± 11.17	<0.001
**1-year**					
PM_10_ (μg/m^3^)	65.76 ± 15.69	56.26 ± 13.74	53.41 ± 10.22	54.86 ± 7.25	<0.001
O_3_ (ppb)	22.79 ± 2.31	23.27 ± 2.92	27.79 ± 2.52	28.66 ± 1.69	<0.001
CO (ppm)	0.779 ± 0.285	0.734 ± 0.251	0.678 ± 0.159	0.627 ± 0.068	<0.001
SO_2_ (ppb)	7.63 ± 4.65	4.52 ± 2.20	3.25 ± 1.56	4.54 ± 0.87	<0.001
NO (ppb)	11.65 ± 9.50	10.96 ± 8.45	7.77 ± 5.85	10.34 ± 2.92	<0.001
NO_2_ (ppb)	22.35 ± 4.91	21.24 ± 5.30	19.03 ± 4.72	20.40 ± 2.68	<0.001
NOx (ppb)	33.87 ± 13.46	32.19 ± 12.96	26.79 ± 9.94	30.74 ± 5.27	<0.001

*CES-D, center for epidemiological studies depression*.

### Individual Pollutants and Depressive Symptoms

**Figure 2:**
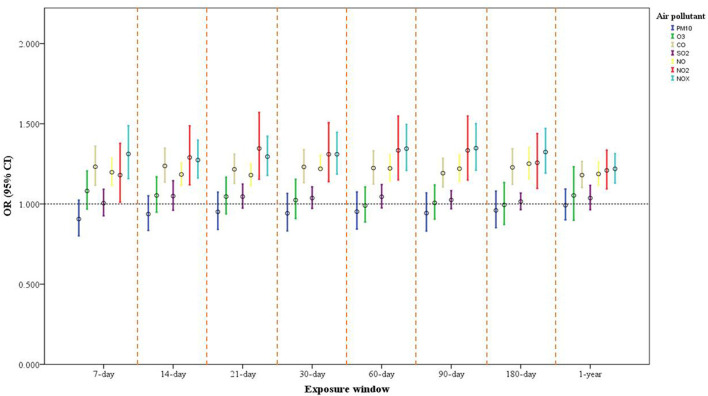
One-pollutant model of air pollution exposure and its association with depressive symptoms in older adults. Model adjusted for sex, age, marital status, educational attainment, financial status (self-reported), physical activity, alcohol intake, heart disease, IADL score, season of study visit, and SPMSQ score. The ORs and 95% CIs for each increase of interquartile range (IQR) in PM_10_, O_3_, CO, SO_2_, NO, NO_2_, and NOx are expressed.

In the single-pollutant model, after covariates were adjusted for, we discovered associations of long- and short-term exposure to CO, NO_2_, NO, and NOx with a heightened risk of depressive symptoms. The odds ratio (OR) and corresponding 95% confidence interval (CI) for each IQR increase in CO in the 7-, 14-, 21-, 30-, 60-, 90-, and 180-day and 1-year moving averages were 1.232 (1.116, 1.361), 1.237 (1.136, 1.348), 1.216 (1.128, 1.311), 1.231 (1.133, 1.338), 1.224 (1.124, 1.332), 1.192 (1.106, 1.285), 1.228 (1.122, 1.344), and 1.180 (1.102, 1.265), respectively. The ORs and 95% CIs for each IQR increment in NOx over the same exposure windows were 1.312 (1.158, 1.488), 1.274 (1.162, 1.398), 1.295 (1.178, 1.423), 1.310 (1.186, 1.447), 1.345 (1.209, 1.496), 1.348 (1.210, 1.501), 1.324 (1.192, 1.471), and 1.219 (1.130, 1.314), respectively. For each IQR increment in NO and NO_2_ over the same exposure windows, the ORs ranged between 1.180 and 1.346 (p < 0.001). Long- and short-term exposure to PM_10_, O_3_, and SO_2_ had no significant association with depressive symptoms (Figure 2 and Supplementary Table S6).

We have categorized the exposures into quartiles to examine the possible non-linear relationships. For example, compared to the Q1 in NOx for 7-day and 1-year exposure window, the Q4 in NOx had the more risk of depressive symptoms (OR = 1.524, 95% CI: 1.215, 1.911 for 7-day exposure window; OR = 1.435, 95% CI: 1.161, 1.773 for 1-year exposure window). Similarly, the Q4 in CO had the more risk of depressive symptoms as compared to the Q1 in CO for 7-day and 1-year exposure window (OR = 1.215, 95% CI: 0.970, 1.522 for 7-day exposure window; OR = 1.348, 95% CI: 1.096, 1.658 for 1-year exposure window) (Supplementary Tables S10–S17).

### Two-Pollutant Exposure Model

**Figure 3:**
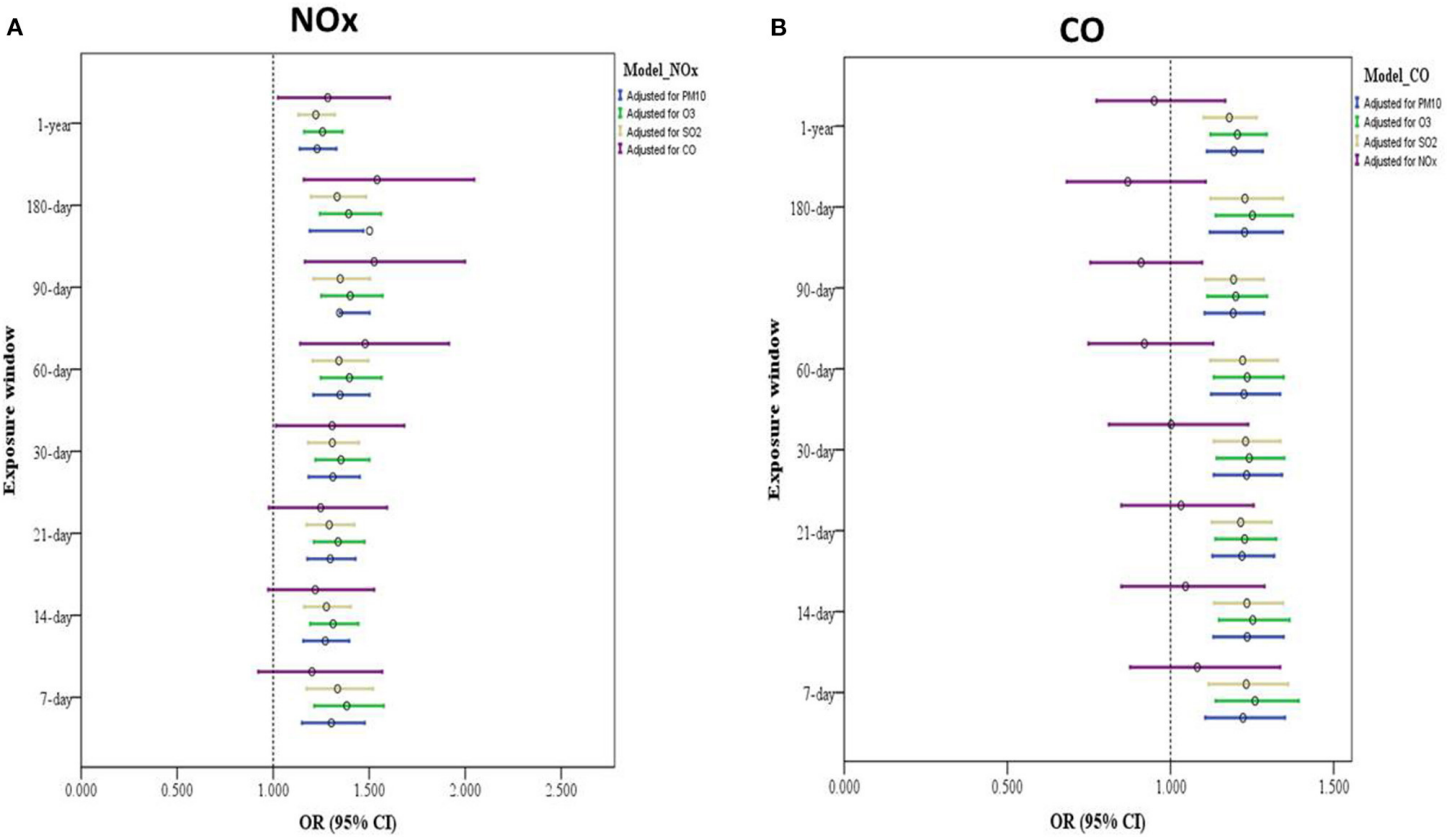
Two-pollutant model of air pollution exposure and its association with depressive symptoms among older adults. **(A)** Model with adjustments for sex, age, educational attainment, marital status, financial status (self-reported), physical activity, alcohol intake, heart disease, IADL score, season of study visit, SPMSQ score, and exposure to other air pollutants (such as PM_10_, O_3_, CO, and SO_2_). **(B)** Model adjusted for sex, marital status, age, educational attainment, financial status (self-reported), physical activity, alcohol intake, heart disease, IADL score, season of study visit, SPMSQ score, and exposure other air pollutants (e.g., NOx, PM_10_, O_3_, and SO_2_).

In the two-pollutant model, we adjusted for the same covariates as we did in the single-pollutant model and added one air pollutant at a time. We discovered associations of long- and short-term exposure to NOx with an increased risk of depressive symptoms after further control for PM_10_, O_3_, or SO_2_ exposure. A significant association was discovered between exposure to NOx (1-year and 30-, 60-, 90-, and 180-day exposure windows) and depressive symptoms after further control for CO exposure (Figure 3A and Supplementary Table S7). Furthermore, the associations of long- and short-term CO exposure with a heightened risk of depressive symptom development after adjusted same covariates and PM_10_, O_3_, or SO_2_ exposure, however, no significant association were found after control for NOx (Figure 3B and Supplementary Table S8).

### Residual Regression Models

In the residual method, the CO exposure was regressed with the NOx exposure. We added the CO residual to the model as the independent variable and adjusted for NOx exposure. We found that CO residuals for the different exposure windows were not associated with the odds of depressive symptoms but Nox for the different exposure windows were positive associated with the odds of symptoms of depression (Supplementary Table S9).

### Joint Effect Analysis

Participants whose cognitive impairment was moderate to severe had higher risks of depressive symptoms with associated with PM_10_ and SO_2_ exposure over various exposure windows (*p* < 0.001; Table 3). However, according to this study, no other air pollutants exerted interaction effects (data not shown).

**Table 3:** Association of interaction of air pollution (selected air pollutants) and cognitive function with depressive symptoms.

**Interaction term**	**Depressive symptoms** ** <10 vs**. **≥10**		***p*-value**
	**OR**	**(95%CI)**	
**PM**_**10**_ * **SPMSQ**^**a**^			
7-day			
PM_10_ * SPMSQ≥3	Ref.		
PM_10_ * SPMSQ <3	1.018	(1.004, 1.032)	0.007
14-day			
PM_10_ * SPMSQ≥3	Ref.		
PM_10_ * SPMSQ <3	1.024	(1.009, 1.039)	0.001
21-day			
PM_10_ * SPMSQ≥3	Ref.		
PM_10_ * SPMSQ <3	1.027	(1.012, 1.042)	<0.001
30-day			
PM_10_ * SPMSQ≥3	Ref.		
PM_10_ * SPMSQ <3	1.026	(1.011, 1.040)	<0.001
60-day			
PM_10_ * SPMSQ≥3	Ref.		
PM_10_ * SPMSQ <3	1.025	(1.012, 1.039)	<0.001
90-day			
PM_10_ * SPMSQ≥3	Ref.		
PM_10_ * SPMSQ <3	1.023	(1.011, 1.036)	<0.001
180-day			
PM_10_ * SPMSQ≥3	Ref.		
PM_10_ * SPMSQ <3	1.021	(1.010, 1.033)	<0.001
1-year			
PM_10_ * SPMSQ≥3	Ref.		
PM_10_ * SPMSQ <3	1.037	(1.014, 1.059)	0.001
**SO**_**2**_ * **SPMSQ**^**b**^			
7-day			
SO_2_ * SPMSQ≥3	Ref.		
SO_2_ * SPMSQ <3	1.122	(1.021, 1.233)	0.016
14-day			
SO_2_ * SPMSQ≥3	Ref.		
SO_2_ * SPMSQ <3	1.131	(1.024, 1.249)	0.014
21 day			
SO_2_ * SPMSQ≥3	Ref.		
SO_2_ * SPMSQ <3	1.126	(1.025, 1.236)	0.012
30-day			
SO_2_ * SPMSQ≥3	Ref.		
SO_2_ * SPMSQ <3	1.132	(1.029, 1.245)	0.010
60-day			
SO_2_ * SPMSQ≥3	Ref.		
SO_2_ * SPMSQ <3	1.135	(1.036, 1.243)	0.006
90-day			
SO_2_ * SPMSQ≥3	Ref.		
SO_2_ * SPMSQ <3	1.116	(1.029, 1.211)	0.007
180-day			
SO_2_ * SPMSQ≥3	Ref.		
SO_2_ * SPMSQ <3	1.095	(1.019, 1.176)	0.012
1-year			
SO_2_ * SPMSQ≥3	Ref.		
SO_2_ * SPMSQ <3	1.089	(0.992, 1.196)	0.073

a *Adjusted for sex, age, marital status, educational attainment, financial status (self-reported), physical activity, alcohol intake, heart disease, IADL score, season of study visit, PM_10_, and SPMSQ score*.

b *Adjusted for sex, age, marital status, educational attainment, financial status (self-reported), physical activity, alcohol intake, heart disease, IADL score, season of study visit, SO_2_, and SPMSQ score*.

*SPMSQ, short portable mental status questionnaire*.

## Discussion

In one-pollutant models, ambient NOx and CO exposure over various exposure windows had a positive association with the development of symptoms of depression by older adults. In two-pollutant and residual regression models, medium- and long-term exposure to ambient NOx still had a significant effect after simultaneous exposure to CO was controlled for. Furthermore, PM_10_ and SO_2_ exposure among older adults with cognitive decline carried a greater risk of depression symptoms than did no cognitive decline. These results demonstrate that long- and short-term air pollutant exposure can increase older adults' risk of depression symptoms, and cognitive decline could render such individuals exposed to air pollution more susceptible to depression.

In a German study, long-term NOx exposure was positively related to depressive symptoms in 821 older women (9). Using repeated measures from 537 South Korean older adults, another study reported an association of short-term NO_2_ exposure with a heightened risk of depressive symptoms (11). The Korean Community Health Survey from 2013 revealed a relationship of long-term NO_2_ and CO exposure with heightened depression risk among individuals aged over 65 years (30). Another study discovered a positive association of long-term NO_2_ exposure with the prescription of antidepressants by using single-exposure regression models based on a large-scale Dutch health survey (31). A study with a nested case–control design revealed relationships of long- and short-term CO exposure with heightened depression risks (32). These studies had similar results to our study. Furthermore, a meta-analysis associated short-term NO_2_ exposure with a heightened depression risk (7). However, unlike the aforementioned studies, we did not identify associations of any other air pollutants (e.g., PM, SO_2_, and O_3_) with the risk of depression in one-pollutant models. These discrepancies could have resulted from differences in exposure profiles, the demographic characteristics of the study population, the exposure assessments used, the outcome assessment scales used, the study design, or our adjustment for confounding factors. Besides, the differences on the effects for the short vs. long term exposures could be due to the different correlations between the same pollutant of the different exposure windows or between the different pollutants of the same exposure window.

Using two-pollutant and residual models, we revealed that medium- and long-term NOx exposure had associations with increased risks of depressive symptoms, after adjustment for simultaneous exposure to CO. However, associations between exposure to CO and depression did not remain significant. This result may be attributable to the strong correlation between NOx and CO (*r* = 0.8 to 0.9) in our study. Similarly, long-term NOx exposure was positively associated with psychopathological symptoms, including internalizing, externalizing, and thought disorder symptoms, after adjustment for simultaneous exposure to PM_2.5_ (33); however, significant associations of PM_2.5_ exposure with psychopathology were not identified in co-pollutant models of NOx and PM_2.5_, because of the high correlation discovered between the two pollutants (*r* = 0.83). One study indicated that ambient gas concentrations can be used as proxies for PM_2.5_ exposure (34). A review revealed that depression and long-term PM_2.5_ exposure were positively associated (5). Taken together, the study results indicate that traffic-related air pollutants could play crucial roles in the development of mental illnesses.

Our results demonstrate cognition-dependent associations of exposure to some air pollutants (e.g., SO_2_ and PM_10_) in the long and short terms with symptoms of depression among older adults. Only one study reported that women with low cognitive ability and exposure to high pollutant (e.g., PM_10_, PM_2.5_, and NOx) concentrations had higher risks of developing depressive symptoms (9). Reports in this field are limited; thus, large-scale investigations are warranted to confirm such findings.

The biological mechanisms of the depression–air pollution association remain unclarified. However, researchers have proposed multiple pathways. First, exposure to ambient air pollution could induce a systemic inflammatory response (35, 36). Systemic inflammation can affect the brain via an induced cytokine response, resulting in neurotoxicity and neuroinflammation (35). Second, air pollutant exposure could increase the levels of hippocampal proinflammatory cytokines and affect hippocampal dendrites, increasing depression-like behavior in animals (37). Third, exposure to air pollutants could cause oxidative stress in the brain (38). Oxidative stress has deleterious effects on dopaminergic neurons, and dopamine depletion in the nervous system is probably related to the neuropathology of depression (39, 40). Fourth, air pollution can negatively affect microvascular endothelial viability in humans and reduce tight junction protein levels, damaging the blood–brain barrier (35). Fifth, ambient air pollutant exposure could disrupt the hypothalamic–pituitary–adrenal axis. This axis regulates stress responses by producing certain hormones (e.g., cortisol). Disruption of this axis is a potential etiological factor of anxiety and depression (41, 42).

The current study was not without limitations. First, the CES-D is not a clinical diagnostic instrument, and it was not designed to assess individuals with chronic mental disorders. However, this screening tool is widely used to evaluate the severity of depressive symptoms, including in populations with somatic or psychiatric disorders (43); it provides cutoffs indicating potential clinical relevance and has high internal consistency, specificity, and sensitivity (20, 44). Second, all participants' residential addresses were not collected, and this may cause measurement errors and result in non-differential misclassification of exposure assessment to attenuated the observed effects. In addition, PM_2.5_ may be strongly correlated with NOx exposure (33); however, we did not assess the effects of PM_2.5_, because it was not measured. Third, unmeasured covariates such as traffic noise and surrounding green areas could have influenced the estimated effects, but related studies have revealed no clear associations of these factors with depression (9, 45).

The nationally representative cohort comprising community-dwelling older adults in Taiwan was a key strength. Our study had robust statistical power for the detection of meaningful associations, and we adjusted for potential confounding factors. Furthermore, the repeated-measures study design enabled the identification of time-dependent associations of exposure with outcomes and helped to minimize the influence of the genetic backgrounds of participants.

In summary, our results indicate that long- and short-term exposure to traffic-associated air pollutants is associated with increased risks of older adults developing depressive symptoms, and air pollution may exacerbate depressive symptoms in older adults with cognitive decline.

## Data Availability Statement

The original contributions presented in the study are included in the article/Supplementary Material, further inquiries can be directed to the corresponding author/s.

## Ethics Statement

The studies involving human participants were reviewed and approved by the Tri-Service General Hospital Institutional Review Board (No.: 2-104-05-153). The patients/participants provided their written informed consent to participate in this study.

## Author Contributions

H-BH: conceived and designed the experiments. K-CW, Y-TL, and H-BH: analyzed the data and wrote the paper. Y-TL, C-CL, Y-YJ, and H-BH: contributed to critical revision of the manuscript. All authors have read and approved the final manuscript.

## Funding

The work was supported by funding from the Ministry of Science and Technology (MOST 105-2314-B-016-005; MOST 107-2813-C-016-030-B; and MOST 110-2628-B-016-003) and Ministry of National Defense-Medical Affairs Bureau (MAB-109-072) in Taiwan, ROC.

## Conflict of Interest

The authors declare that the research was conducted in the absence of any commercial or financial relationships that could be construed as a potential conflict of interest.

## Publisher's Note

All claims expressed in this article are solely those of the authors and do not necessarily represent those of their affiliated organizations, or those of the publisher, the editors and the reviewers. Any product that may be evaluated in this article, or claim that may be made by its manufacturer, is not guaranteed or endorsed by the publisher.
