# Supplementary material for: Associations Between Symptoms of Depression and Air Pollutant Exposure Among Older Adults: Results From the Taiwan Longitudinal Study on Aging (TLSA)
Source: Front Public Health. 2022 Jan 12;9:779192. doi: 10.3389/fpubh.2021.779192 (PMC8790292; doi:10.3389/fpubh.2021.779192)
Supplement: Supplementary file 1 [file Data_Sheet_1.docx]

Supplementary Material

**Associations between symptoms of depression and air pollutant exposure among older adults: Results from the Taiwan longitudinal study on aging (TLSA)**

Kuan-Chin Wang, Yuan-Ting C. Lo, Chun-Cheng Liao, Yann-Yuh Jou, Han-Bin Huang

###
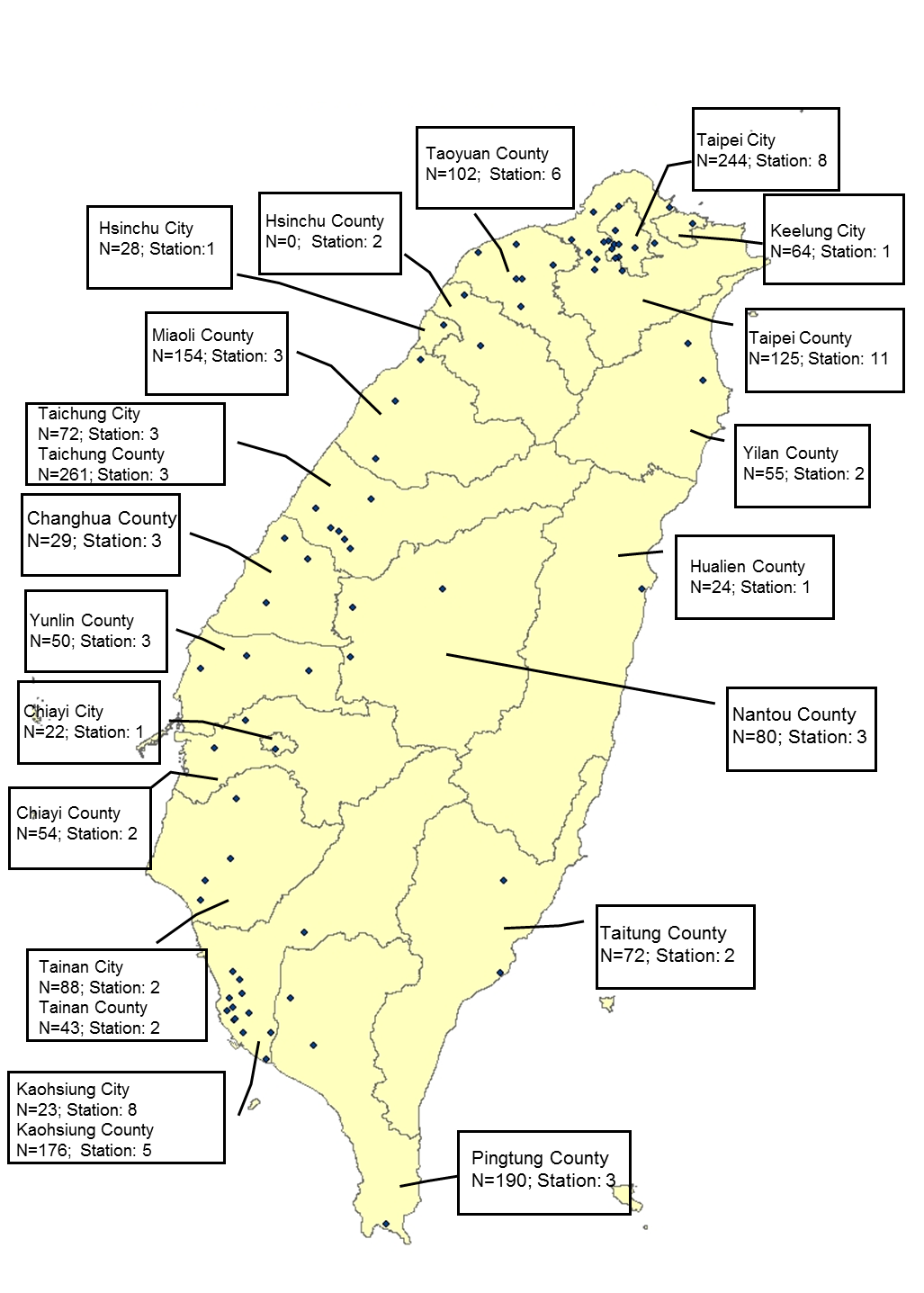


**Supplementary Figure S1.** The distribution of study population (N=1956) in 1996 and monitoring stations in each city/county in Taiwan.

**Supplementary Table S1.** Assessment of depression measures in the present study.

|  | Rarely or none of the time | Some of the time | Occasionally | Most of the time |
| --- | --- | --- | --- | --- |
| **Center for Epidemiological Studies –Depression (CESD-10)** |  |  |  |  |
| ***(1)*** During the past week I did not feel like eating; my appetite was poor. | 0 | 1 | 2 | 3 |
| ***(2)*** During the past week I felt that everything I did was an effort. | 0 | 1 | 2 | 3 |
| ***(3)*** During the past week my sleep was restless | 0 | 1 | 2 | 3 |
| ***(4)*** During the past week I was happy. | 3 | 2 | 1 | 0 |
| ***(5)*** During the past week I felt lonely | 0 | 1 | 2 | 3 |
| ***(6)*** During the past week people were unfriendly. | 0 | 1 | 2 | 3 |
| ***(7)*** During the past week I enjoyed life. | 3 | 2 | 1 | 0 |
| ***(8)*** During the past week I felt sad. | 0 | 1 | 2 | 3 |
| ***(9)*** During the past week I felt depressed. | 0 | 1 | 2 | 3 |
| ***(10)*** During the past week I could not get "going." | 0 | 1 | 2 | 3 |

**Supplementary** **Table S2**. Distribution of air pollutant concentrations over various exposure windows for each survey year

| Variable | Year 1996 (N=1,956) | Year 1999 (N=1,699) | Year 2003 (N=1,248) | Year 2007(N=873) | *P* for trend |
| --- | --- | --- | --- | --- | --- |
|  | Mean±SD | Mean±SD | Mean±SD | Mean±SD |  |
|  |  |  |  |  |  |
| 14-day |  |  |  |  |  |
| PM_10_ (µg/m^3^) | 68.72±15.16 | 70.11±22.50 | 52.63±18.10 | 49.12±19.15 | <0.001 |
| O_3_ (ppb) | 30.67±3.15 | 29.90±4.61 | 31.44±6.75 | 30.01±9.59 | <0.001 |
| CO (ppm) | 0.818±0.266 | 0.722±0.235 | 0.634±0.130 | 0.482±0.171 | <0.001 |
| SO_2_ (ppb) | 6.52±3.52 | 5.09±2.82 | 2.89±1.54 | 4.03±1.29 | <0.001 |
| NO (ppb) | 9.84±8.53 | 10.19±8.46 | 6.18±4.88 | 6.42±5.02 | <0.001 |
| NO_2_ (ppb) | 24.38±5.74 | 24.05±5.93 | 19.34±5.41 | 15.99±6.72 | <0.001 |
| NOx (ppb) | 34.28±13.20 | 34.18±13.44 | 26.13±9.43 | 22.41±10.85 | <0.001 |
| 21-day |  |  |  |  |  |
| PM_10_ (µg/m^3^) | 71.38±20.16 | 66.81±19.93 | 52.11±17.21 | 50.09±18.08 | <0.001 |
| O_3_ (ppb) | 29.30±3.50 | 26.85±3.92 | 31.36±6.35 | 30.44±8.89 | <0.001 |
| CO (ppm) | 0.823±0.285 | 0.774±0.277 | 0.629±0.124 | 0.489±0.168 | <0.001 |
| SO_2_(ppb) | 6.80±3.93 | 5.16±2.78 | 2.83±1.47 | 4.06±1.28 | <0.001 |
| NO (ppb) | 10.15±8.90 | 12.16±10.43 | 6.07±4.73 | 6.41±4.93 | <0.001 |
| NO_2_ (ppb) | 25.22±5.86 | 24.98±6.45 | 18.92±5.26 | 16.27±6.67 | <0.001 |
| NOx (ppb) | 35.39±13.59 | 37.09±15.96 | 24.96±8.86 | 22.68±10.75 | <0.001 |
| 30-day |  |  |  |  |  |
| PM_10_ (µg/m^3^) | 68.61±21.26 | 66.30±22.27 | 50.86±16.23 | 51.42±16.84 | <0.001 |
| O3 (ppb) | 27.42±3.62 | 25.84±4.43 | 30.91±6.15 | 30.84±7.99 | <0.001 |
| CO (ppm) | 0.809±0.278 | 0.777±0.254 | 0.620±0.123 | 0.499±0.165 | <0.001 |
| SO_2_ (ppb) | 6.61±3.8 | 4.97±2.71 | 2.77±1.40 | 4.10±1.29 | <0.001 |
| NO (ppb) | 10.57±8.88 | 12.07±9.56 | 5.95±4.56 | 6.43±4.89 | <0.001 |
| NO_2_ (ppb) | 24.99±5.70 | 25.30±6.26 | 18.34±5.11 | 16.67±6.66 | <0.001 |
| NOx (ppb) | 35.53±13.47 | 37.32±14.94 | 24.27±8.58 | 23.11±10.75 | <0.001 |
| 60-day |  |  |  |  |  |
| PM_10_ (µg/m^3^) | 74.96±23.43 | 70.55±23.48 | 45.91±13.38 | 55.78±13.31 | <0.001 |
| O_3_ (ppb) | 26.33±4.03 | 27.60±4.53 | 28.51±5.38 | 31.78±4.84 | <0.001 |
| CO (ppm) | 0.830±0.298 | 0.763±0.229 | 0.592±0.120 | 0.533±0.155 | <0.001 |
| SO_2_ (ppb) | 7.30±4.00 | 4.94±2.67 | 2.56±1.25 | 4.26±1.31 | <0.001 |
| NO (ppb) | 11.23±9.64 | 11.94±9.20 | 5.88±4.49 | 6.61±4.58 | <0.001 |
| NO_2_ (ppb) | 25.35±5.71 | 25.31±5.86 | 16.74±4.75 | 18.03±6.40 | <0.001 |
| NOx (ppb) | 36.50±14.12 | 37.23±13.92 | 22.61±8.24 | 24.64±10.42 | <0.001 |
| 90-day |  |  |  |  |  |
| PM_10_ (µg/m^3^) | 71.58±22.11 | 73.04±24.61 | 42.56±10.99 | 58.36±12.16 | <0.001 |
| O_3_ (ppb) | 25.88±3.92 | 25.93±4.42 | 26.50±4.37 | 31.69±3.34 | <0.001 |
| CO (ppm) | 0.786±0.291 | 0.766±0.218 | 0.574±0.124 | 0.558±0.147 | <0.001 |
| SO_2_ (ppb) | 7.46±4.72 | 5.10±2.97 | 2.48±1.15 | 4.33±1.34 | <0.001 |
| NO (ppb) | 11.27±10.04 | 12.60±8.98 | 5.89±4.50 | 6.98±4.61 | <0.001 |
| NO_2_ (ppb) | 24.65±5.45 | 25.48±5.73 | 15.78±4.45 | 18.88±6.10 | <0.001 |
| NOx (ppb) | 35.86±14.32 | 38.06±13.50 | 21.66±8.15 | 25.86±10.28 | <0.001 |

**Supplementary** **Table S3**. Distribution of air pollutant concentrations over various exposure windows during 1996 to 2007

| Variable | P25 | P50 | P75 | IQR |
| --- | --- | --- | --- | --- |
|  |  |  |  |  |
| 7-day |  |  |  |  |
| PM_10_ (µg/m^3^) | 42.29 | 60.11 | 74.42 | 32.13 |
| O_3_ (ppb) | 27.80 | 33.19 | 35.90 | 8.1 |
| CO (ppm) | 0.51 | 0.67 | 0.77 | 0.26 |
| SO_2_ (ppb) | 2.70 | 4.34 | 6.15 | 3.45 |
| NO (ppb) | 3.10 | 6.28 | 9.71 | 6.61 |
| NO_2_ (ppb) | 16.30 | 24.05 | 27.70 | 11.4 |
| NOx (ppb) | 19.19 | 29.89 | 37.27 | 18.08 |
| 14-day |  |  |  |  |
| PM_10_ (µg/m^3^) | 46.69 | 66.47 | 73.89 | 27.2 |
| O_3_ (ppb) | 26.97 | 29.95 | 33.25 | 6.28 |
| CO (ppm) | 0.54 | 0.67 | 0.80 | 0.26 |
| SO_2_ (ppb) | 2.84 | 4.22 | 6.24 | 3.4 |
| NO (ppb) | 3.55 | 6.76 | 9.52 | 5.97 |
| NO_2_ (ppb) | 16.60 | 23.21 | 26.83 | 10.23 |
| NOx (ppb) | 20.44 | 30.64 | 35.05 | 14.61 |
| 21-day |  |  |  |  |
| PM_10_ (µg/m^3^) | 46.50 | 63.23 | 76.64 | 30.14 |
| O_3_ (ppb) | 25.72 | 28.18 | 32.10 | 6.38 |
| CO (ppm) | 0.54 | 0.71 | 0.79 | 0.25 |
| SO_2_ (ppb) | 3.06 | 4.18 | 6.05 | 2.99 |
| NO (ppb) | 3.68 | 7.21 | 10.11 | 6.43 |
| NO_2_ (ppb) | 16.71 | 23.59 | 28.26 | 11.55 |
| NOx (ppb) | 20.58 | 31.62 | 36.98 | 16.4 |
| 30-day |  |  |  |  |
| PM_10_ (µg/m^3^) | 43.37 | 60.23 | 75.57 | 32.2 |
| O_3_ (ppb) | 24.26 | 26.79 | 31.41 | 7.15 |
| CO (ppm) | 0.54 | 0.69 | 0.80 | 0.26 |
| SO_2_ (ppb) | 3.04 | 4.18 | 5.68 | 2.64 |
| NO (ppb) | 3.60 | 7.46 | 10.93 | 7.33 |
| NO_2_ (ppb) | 17.44 | 23.70 | 27.67 | 10.23 |
| NOx (ppb) | 20.91 | 30.99 | 37.49 | 16.58 |
| 60-day |  |  |  |  |
| PM_10_ (µg/m^3^) | 46.62 | 61.03 | 80.18 | 33.56 |
| O_3_ (ppb) | 24.31 | 27.27 | 30.78 | 6.47 |
| CO (ppm) | 0.54 | 0.69 | 0.81 | 0.27 |
| SO_2_ (ppb) | 3.21 | 4.40 | 6.16 | 2.95 |
| NO (ppb) | 3.75 | 7.86 | 11.24 | 7.49 |
| NO_2_ (ppb) | 17.56 | 23.68 | 28.05 | 10.49 |
| NOx (ppb) | 21.13 | 31.92 | 38.66 | 17.53 |

**Supplementary** **Table S3**. (cont.)

| Variable | P25 | P50 | P75 | IQR |
| --- | --- | --- | --- | --- |
|  |  |  |  |  |
| 90-day |  |  |  |  |
| PM_10_ (µg/m^3^) | 46.06 | 59.29 | 80.02 | 33.96 |
| O_3_ (ppb) | 23.70 | 26.07 | 29.42 | 5.72 |
| CO (ppm) | 0.54 | 0.68 | 0.77 | 0.23 |
| SO_2_ (ppb) | 3.19 | 4.50 | 5.87 | 2.68 |
| NO (ppb) | 3.87 | 8.08 | 11.53 | 7.66 |
| NO_2_ (ppb) | 17.29 | 23.23 | 27.45 | 10.16 |
| NOx (ppb) | 20.97 | 32.72 | 38.66 | 17.69 |
| 180-day |  |  |  |  |
| PM_10_ (µg/m^3^) | 46.32 | 61.46 | 80.62 | 34.3 |
| O_3_ (ppb) | 22.91 | 25.97 | 28.89 | 5.98 |
| CO (ppm) | 0.55 | 0.69 | 0.82 | 0.27 |
| SO_2_ (ppb) | 2.98 | 4.33 | 6.08 | 3.1 |
| NO (ppb) | 4.22 | 8.91 | 13.05 | 8.83 |
| NO_2_ (ppb) | 17.74 | 23.20 | 26.99 | 9.25 |
| NOx (ppb) | 22.43 | 33.05 | 39.39 | 16.96 |
| 1-year |  |  |  |  |
| PM_10_ (µg/m^3^) | 49.33 | 55.74 | 66.87 | 17.54 |
| O_3_ (ppb) | 22.35 | 24.58 | 27.76 | 5.41 |
| CO (ppm) | 0.56 | 0.68 | 0.78 | 0.22 |
| SO_2_ (ppb) | 3.36 | 4.39 | 6.84 | 3.48 |
| NO (ppb) | 5.45 | 7.78 | 12.10 | 6.65 |
| NO_2_ (ppb) | 17.89 | 22.10 | 24.10 | 6.21 |
| NOx (ppb) | 23.17 | 30.86 | 35.09 | 11.92 |

**Supplementary** **Table S4**. Pearson correlation of the same air pollutant over various exposure windows during survey period

|  | 7-day | 14-day | 21-day | 30-day | 60-day | 90-day | 180-day | 1-year |
| --- | --- | --- | --- | --- | --- | --- | --- | --- |
| PM_10_ |  |  |  |  |  |  |  |  |
| 7-day | 1 |  |  |  |  |  |  |  |
| 14-day | 0.948^✽✽^ | 1 |  |  |  |  |  |  |
| 21-day | 0.876^✽✽^ | 0.964^✽✽^ | 1 |  |  |  |  |  |
| 30-day | 0.865^✽✽^ | 0.943^✽✽^ | 0.984^✽✽^ | 1 |  |  |  |  |
| 60-day | 0.801^✽✽^ | 0.895^✽✽^ | 0.944^✽✽^ | 0.965^✽✽^ | 1 |  |  |  |
| 90-day | 0.791^✽✽^ | 0.871^✽✽^ | 0.907^✽✽^ | 0.936^✽✽^ | 0.982^✽✽^ | 1 |  |  |
| 180-day | 0.707^✽✽^ | 0.794^✽✽^ | 0.857^✽✽^ | 0.883^✽✽^ | 0.939^✽✽^ | 0.958^✽✽^ | 1 |  |
| 1-year | 0.693^✽✽^ | 0.780^✽✽^ | 0.858^✽✽^ | 0.862v | 0.868^✽✽^ | 0.838^✽✽^ | 0.909^✽✽^ | 1 |
| O_3_ |  |  |  |  |  |  |  |  |
| 7-day | 1 |  |  |  |  |  |  |  |
| 14-day | 0.900^✽✽^ | 1 |  |  |  |  |  |  |
| 21-day | 0.750^✽✽^ | 0.924^✽✽^ | 1 |  |  |  |  |  |
| 30-day | 0.667^✽✽^ | 0.840^✽✽^ | 0.951^✽✽^ | 1 |  |  |  |  |
| 60-day | 0.431^✽✽^ | 0.587^✽✽^ | 0.693^✽✽^ | 0.808^✽✽^ | 1 |  |  |  |
| 90-day | 0.383^✽✽^ | 0.512^✽✽^ | 0.621^✽✽^ | 0.729^✽✽^ | 0.948^✽✽^ | 1 |  |  |
| 180-day | 0.347^✽✽^ | 0.487^✽✽^ | 0.579^✽✽^ | 0.666^✽✽^ | 0.845^✽✽^ | 0.890^✽✽^ | 1 |  |
| 1-year | 0.221^✽✽^ | 0.403^✽✽^ | 0.538^✽✽^ | 0.627^✽✽^ | 0.682^✽✽^ | 0.699^✽✽^ | 0.850^✽✽^ | 1 |
| CO |  |  |  |  |  |  |  |  |
| 7-day | 1 |  |  |  |  |  |  |  |
| 14-day | 0.964^✽✽^ | 1 |  |  |  |  |  |  |
| 21-day | 0.938^✽✽^ | 0.989^✽✽^ | 1 |  |  |  |  |  |
| 30-day | 0.936^✽✽^ | 0.983^✽✽^ | 0.996^✽✽^ | 1 |  |  |  |  |
| 60-day | 0.919^✽✽^ | 0.971^✽✽^ | 0.979^✽✽^ | 0.987^✽✽^ | 1 |  |  |  |
| 90-day | 0.901^✽✽^ | 0.949^✽✽^ | 0.963^✽✽^ | 0.976^✽✽^ | 0.992^✽✽^ | 1 |  |  |
| 180-day | 0.887^✽✽^ | 0.921^✽✽^ | 0.928^✽✽^ | 0.941^✽✽^ | 0.969^✽✽^ | 0.978^✽✽^ | 1 |  |
| 1-year | 0.852^✽✽^ | 0.908^✽✽^ | 0.922^✽✽^ | 0.922^✽✽^ | 0.929^✽✽^ | 0.933^✽✽^ | 0.943^✽✽^ | 1 |

**Supplementary** **Table S4**. (cont.)

|  | 7-day | 14-day | 21-day | 30-day | 60-day | 90-day | 180-day | 1-year |
| --- | --- | --- | --- | --- | --- | --- | --- | --- |
| SO_2_ |  |  |  |  |  |  |  |  |
| 7-day | 1 |  |  |  |  |  |  |  |
| 14-day | 0.929^✽✽^ | 1 |  |  |  |  |  |  |
| 21-day | 0.904^✽✽^ | 0.988^✽✽^ | 1 |  |  |  |  |  |
| 30-day | 0.901^✽✽^ | 0.972^✽✽^ | 0.994^✽✽^ | 1 |  |  |  |  |
| 60-day | 0.877^✽✽^ | 0.944^✽✽^ | 0.975^✽✽^ | 0.988^✽✽^ | 1 |  |  |  |
| 90-day | 0.908^✽✽^ | 0.921^✽✽^ | 0.948^✽✽^ | 0.965^✽✽^ | 0.980^✽✽^ | 1 |  |  |
| 180-day | 0.901^✽✽^ | 0.851^✽✽^ | 0.873^✽✽^ | 0.890^✽✽^ | 0.915^✽✽^ | 0.971^✽✽^ | 1 |  |
| 1-year | 0.895^✽✽^ | 0.860^✽✽^ | 0.864^✽✽^ | 0.868^✽✽^ | 0.888^✽✽^ | 0.936^✽✽^ | 0.966^✽✽^ | 1 |
| NO |  |  |  |  |  |  |  |  |
| 7-day | 1 |  |  |  |  |  |  |  |
| 14-day | 0.968^✽✽^ | 1 |  |  |  |  |  |  |
| 21-day | 0.938^✽✽^ | 0.989^✽✽^ | 1 |  |  |  |  |  |
| 30-day | 0.941^✽✽^ | 0.985^✽✽^ | 0.995^✽✽^ | 1 |  |  |  |  |
| 60-day | 0.938^✽✽^ | 0.978^✽✽^ | 0.983^✽✽^ | 0.991^✽✽^ | 1 |  |  |  |
| 90-day | 0.936^✽✽^ | 0.971^✽✽^ | 0.977^✽✽^ | 0.987^✽✽^ | 0.996^✽✽^ | 1 |  |  |
| 180-day | 0.939^✽✽^ | 0.951^✽✽^ | 0.942^✽✽^ | 0.958^✽✽^ | 0.976^✽✽^ | 0.981^✽✽^ | 1 |  |
| 1-year | 0.917^✽✽^ | 0.941^✽✽^ | 0.933^✽✽^ | 0.938^✽✽^ | 0.952^✽✽^ | 0.949^✽✽^ | 0.967^✽✽^ | 1 |
| NO_2_ |  |  |  |  |  |  |  |  |
| 7-day | 1 |  |  |  |  |  |  |  |
| 14-day | 0.957^✽✽^ | 1 |  |  |  |  |  |  |
| 21-day | 0.935^✽✽^ | 0.990^✽✽^ | 1 |  |  |  |  |  |
| 30-day | 0.924^✽✽^ | 0.979^✽✽^ | 0.993^✽✽^ | 1 |  |  |  |  |
| 60-day | 0.885^✽✽^ | 0.942^✽✽^ | 0.963^✽✽^ | 0.979^✽✽^ | 1 |  |  |  |
| 90-day | 0.852^✽✽^ | 0.901^✽✽^ | 0.926^✽✽^ | 0.950^✽✽^ | 0.987^✽✽^ | 1 |  |  |
| 180-day | 0.792^✽✽^ | 0.828^✽✽^ | 0.854^✽✽^ | 0.878^✽✽^ | 0.937^✽✽^ | 0.966^✽✽^ | 1 |  |
| 1-year | 0.758^✽✽^ | 0.842^✽✽^ | 0.846^✽✽^ | 0.843^✽✽^ | 0.851^✽✽^ | 0.838^✽✽^ | 0.847^✽✽^ | 1 |

**Supplementary** **Table S4**.(cont.)

|  | 7-day | 14-day | 21-day | 30-day | 60-day | 90-day | 180-day | 1-year |
| --- | --- | --- | --- | --- | --- | --- | --- | --- |
| NOx |  |  |  |  |  |  |  |  |
| 7-day | 1 |  |  |  |  |  |  |  |
| 14-day | 0.967^✽✽^ | 1 |  |  |  |  |  |  |
| 21-day | 0.943^✽✽^ | 0.991^✽✽^ | 1 |  |  |  |  |  |
| 30-day | 0.940^✽✽^ | 0.985^✽✽^ | 0.995^✽✽^ | 1 |  |  |  |  |
| 60-day | 0.924^✽✽^ | 0.972^✽✽^ | 0.981^✽✽^ | 0.990^✽✽^ | 1 |  |  |  |
| 90-day | 0.910^✽✽^ | 0.945^✽✽^ | 0.966^✽✽^ | 0.979^✽✽^ | 0.993^✽✽^ | 1 |  |  |
| 180-day | 0.887^✽✽^ | 0.915^✽✽^ | 0.918^✽✽^ | 0.936^✽✽^ | 0.965^✽✽^ | 0.977^✽✽^ | 1 |  |
| 1-year | 0.848^✽✽^ | 0.904^✽✽^ | 0.901^✽✽^ | 0.899^✽✽^ | 0.912^✽✽^ | 0.906^✽✽^ | 0.919^✽✽^ | 1 |
|  |  |  |  |  |  |  |  |  |

^✽✽^ *P* <0.01

**Supplementary** **Table S5**. Pearson correlation of the different air pollutants for the same exposure windows during survey period

|  | PM_10_(µg/m^3^) | O_3_(ppb) | CO(ppm) | SO_2_(ppb) | NO(ppb) | NO_2_(ppb) | NOx(ppb) |
| --- | --- | --- | --- | --- | --- | --- | --- |
| 7-day |  |  |  |  |  |  |  |
| PM_10_(µg/m^3^) | 1 |  |  |  |  |  |  |
| O_3_(ppb) | 0.313^✽✽^ | 1 |  |  |  |  |  |
| CO(ppm) | 0.071^✽✽^ | -0.020 | 1 |  |  |  |  |
| SO_2_(ppb) | 0.469^✽✽^ | 0.114^✽✽^ | 0.136^✽✽^ | 1 |  |  |  |
| NO(ppb) | -0.247^✽✽^ | -0.173^✽✽^ | 0.828^✽✽^ | 0.130^✽✽^ | 1 |  |  |
| NO_2_(ppb) | 0.394^✽✽^ | -0.069^✽✽^ | 0.754^✽✽^ | 0.483^✽✽^ | 0.558^✽✽^ | 1 |  |
| NOx(ppb) | 0.076^✽✽^ | -0.136^✽✽^ | 0.897^✽✽^ | 0.345^✽✽^ | 0.888^✽✽^ | 0.877^✽✽^ | 1 |
| 14-day |  |  |  |  |  |  |  |
| PM_10_(µg/m^3^) | 1 |  |  |  |  |  |  |
| O_3_(ppb) | 0.236^✽✽^ | 1 |  |  |  |  |  |
| CO(ppm) | 0.085^✽✽^ | -0.072^✽✽^ | 1 |  |  |  |  |
| SO_2_(ppb) | 0.514^✽✽^ | 0.028^✽✽^ | 0.220^✽✽^ | 1 |  |  |  |
| NO(ppb) | -0.194^✽✽^ | -0.199^✽✽^ | 0.868^✽✽^ | 0.165^✽✽^ | 1 |  |  |
| NO_2_(ppb) | 0.342^✽✽^ | -0.135^✽✽^ | 0.809^✽✽^ | 0.497^✽✽^ | 0.676^✽✽^ | 1 |  |
| NOx(ppb) | 0.063^✽✽^ | -0.184^✽✽^ | 0.919^✽✽^ | 0.350^✽✽^ | 0.926^✽✽^ | 0.904^✽✽^ | 1 |
| 21-day |  |  |  |  |  |  |  |
| PM_10_(µg/m^3^) | 1 |  |  |  |  |  |  |
| O_3_(ppb) | 0.120^✽✽^ | 1 |  |  |  |  |  |
| CO(ppm) | 0.048^✽✽^ | -0.163^✽✽^ | 1 |  |  |  |  |
| SO_2_(ppb) | 0.560^✽✽^ | -0.033^✽✽^ | 0.190^✽✽^ | 1 |  |  |  |
| NO(ppb) | -0.199^✽✽^ | -0.250^✽✽^ | 0.893^✽✽^ | 0.146^✽✽^ | 1 |  |  |
| NO_2_(ppb) | 0.329^✽✽^ | -0.257^✽✽^ | 0.813^✽✽^ | 0.494^✽✽^ | 0.696^✽✽^ | 1 |  |
| NOx(ppb) | 0.042^✽✽^ | -0.274^✽✽^ | 0.930^✽✽^ | 0.329^✽✽^ | 0.936^✽✽^ | 0.904^✽✽^ | 1 |

**Supplementary** **Table S5**. (cont.)

|  | PM_10_(µg/m^3^) | O_3_(ppb) | CO(ppm) | SO_2_(ppb) | NO(ppb) | NO_2_(ppb) | NOx(ppb) |
| --- | --- | --- | --- | --- | --- | --- | --- |
| 30-day |  |  |  |  |  |  |  |
| PM_10_(µg/m^3^) | 1 |  |  |  |  |  |  |
| O_3_(ppb) | 0.017 | 1 |  |  |  |  |  |
| CO(ppm) | 0.023 | -0.231^✽✽^ | 1 |  |  |  |  |
| SO_2_(ppb) | 0.558^✽✽^ | -0.125^✽✽^ | 0.173^✽✽^ | 1 |  |  |  |
| NO(ppb) | -0.197^✽✽^ | -0.291^✽✽^ | 0.900^✽✽^ | 0.149^✽✽^ | 1 |  |  |
| NO_2_(ppb) | 0.301^✽✽^ | -0.352^✽✽^ | 0.815^✽✽^ | 0.473^✽✽^ | 0.716^✽✽^ | 1 |  |
| NOx(ppb) | 0.033^✽^ | -0.344^✽✽^ | 0.929^✽✽^ | 0.321^✽✽^ | 0.939^✽✽^ | 0.913^✽✽^ | 1 |
| 60-day |  |  |  |  |  |  |  |
| PM_10_(µg/m^3^) | 1 |  |  |  |  |  |  |
| O_3_(ppb) | 0.045^✽✽^ | 1 |  |  |  |  |  |
| CO(ppm) | 0.065^✽✽^ | -0.295^✽✽^ | 1 |  |  |  |  |
| SO_2_(ppb) | 0.621^✽✽^ | -0.137^✽✽^ | 0.227^✽✽^ | 1 |  |  |  |
| NO(ppb) | -0.157^✽✽^ | -0.306^✽✽^ | 0.902^✽✽^ | 0.166^✽✽^ | 1 |  |  |
| NO_2_(ppb) | 0.393^✽✽^ | -0.373^✽✽^ | 0.799^✽✽^ | 0.524^✽✽^ | 0.704^✽✽^ | 1 |  |
| NOx(ppb) | 0.100^✽✽^ | -0.365^✽✽^ | 0.925^✽✽^ | 0.355^✽✽^ | 0.937^✽✽^ | 0.908^✽✽^ | 1 |
| 90-day |  |  |  |  |  |  |  |
| PM_10_(µg/m^3^) | 1 |  |  |  |  |  |  |
| O_3_(ppb) | 0.033^✽^ | 1 |  |  |  |  |  |
| CO(ppm) | 0.055^✽✽^ | -0.272^✽✽^ | 1 |  |  |  |  |
| SO_2_(ppb) | 0.561^✽✽^ | -0.151^✽✽^ | 0.150^✽✽^ | 1 |  |  |  |
| NO(ppb) | -0.120^✽✽^ | -0.268^✽✽^ | 0.919^✽✽^ | 0.162^✽✽^ | 1 |  |  |
| NO_2_(ppb) | 0.438^✽✽^ | -0.363^✽✽^ | 0.780^✽✽^ | 0.505^✽✽^ | 0.703^✽✽^ | 1 |  |
| NOx(ppb) | 0.137^✽✽^ | -0.336^✽✽^ | 0.927^✽✽^ | 0.339^✽✽^ | 0.940^✽✽^ | 0.904^✽✽^ | 1 |

**Supplementary** **Table S5**. (cont.)

|  | PM_10_(µg/m^3^) | O_3_(ppb) | CO(ppm) | SO_2_(ppb) | NO(ppb) | NO_2_(ppb) | NOx(ppb) |
| --- | --- | --- | --- | --- | --- | --- | --- |
| 180-day |  |  |  |  |  |  |  |
| PM_10_(µg/m^3^) | 1 |  |  |  |  |  |  |
| O_3_(ppb) | 0.029^✽^ | 1 |  |  |  |  |  |
| CO(ppm) | 0.055^✽✽^ | -0.389^✽✽^ | 1 |  |  |  |  |
| SO_2_(ppb) | 0.519^✽✽^ | -0.198^✽✽^ | 0.113^✽✽^ | 1 |  |  |  |
| NO(ppb) | -0.127^✽✽^ | -0.353^✽✽^ | 0.916^✽✽^ | 0.177^✽✽^ | 1 |  |  |
| NO_2_(ppb) | 0.478^✽✽^ | -0.479^✽✽^ | 0.763^✽✽^ | 0.496^✽✽^ | 0.667^✽✽^ | 1 |  |
| NOx(ppb) | 0.147^✽✽^ | -0.445^✽✽^ | 0.929^✽✽^ | 0.345^✽✽^ | 0.936^✽✽^ | 0.887^✽✽^ |  |
| 1-year |  |  |  |  |  |  |  |
| PM_10_(µg/m^3^) | 1 |  |  |  |  |  |  |
| O_3_(ppb) | -0.011 | 1 |  |  |  |  |  |
| CO(ppm) | -0.201^✽✽^ | -0.347^✽✽^ | 1 |  |  |  |  |
| SO_2_(ppb) | 0.435^✽✽^ | -0.258^✽✽^ | 0.073^✽✽^ | 1 |  |  |  |
| NO(ppb) | -0.307^✽✽^ | -0.278^✽✽^ | 0.926^✽✽^ | 0.189^✽✽^ | 1 |  |  |
| NO_2_(ppb) | 0.110^✽✽^ | -0.409^✽✽^ | 0.790^✽✽^ | 0.396^✽✽^ | 0.751^✽✽^ | 1 |  |
| NOx(ppb) | -0.160^✽✽^ | -0.348^✽✽^ | 0.930^✽✽^ | 0.284^✽✽^ | 0.963^✽✽^ | 0.900^✽✽^ | 1 |

^✽✽^ *P* <0.01

^✽^ *P* <0.05

**Supplementary** **Table S6**. One-pollutant model of air pollution exposure and its association with depressive symptoms in older adults.

| Exposure window | | CESD  <10 VS. ≥10 | | |
| --- | --- | --- | --- | --- |
|  |  | AOR ^a^ | 95%CI | p-value |
| 7-day | | | | |
|  | PM_10_ (µg/m^3^) | 0.906 | 0.801,1.024 | 0.115 |
|  | O_3_ (ppb) | 1.081 | 0.968,1.206 | 0.163 |
|  | CO (ppm) | 1.232 | 1.116,1.361 | <0.001 |
|  | SO_2_ (ppb) | 1.005 | 0.926,1.092 | 0.887 |
|  | NO (ppb) | 1.198 | 1.116,1.285 | <0.001 |
|  | NO_2_ (ppb) | 1.180 | 1.011,1.378 | 0.035 |
|  | NO_X_ (ppb) | 1.312 | 1.158,1.488 | <0.001 |
| 14-day | | | | |
|  | PM_10_ (µg/m^3^) | 0.937 | 0.835,1.051 | 0.269 |
|  | O_3_ (ppb) | 1.053 | 0.949,1.169 | 0.327 |
|  | CO (ppm) | 1.237 | 1.136,1.348 | <0.001 |
|  | SO_2_ (ppb) | 1.049 | 0.961,1.145 | 0.281 |
|  | NO (ppb) | 1.184 | 1.115,1.256 | <0.001 |
|  | NO_2_ (ppb) | 1.290 | 1.119,1.487 | <0.001 |
|  | NO_X_ (ppb) | 1.274 | 1.162,1.398 | <0.001 |
| 21-day | | | | |
|  | PM_10_ (µg/m^3^) | 0.951 | 0.841,1.074 | 0.422 |
|  | O_3_ (ppb) | 1.046 | 0.938,1.167 | 0.416 |
|  | CO (ppm) | 1.216 | 1.128,1.311 | <0.001 |
|  | SO_2_ (ppb) | 1.046 | 0.974,1.124 | 0.211 |
|  | NO (ppb) | 1.180 | 1.115,1.249 | <0.001 |
|  | NO_2_ (ppb) | 1.346 | 1.154,1.570 | <0.001 |
|  | NO_X_ (ppb) | 1.295 | 1.178,1.423 | <0.001 |
| 30-day | | | | |
|  | PM_10_ (µg/m^3^) | 0.942 | 0.832,1.066 | 0.348 |
|  | O_3_ (ppb) | 1.024 | 0.909,1.154 | 0.692 |
|  | CO (ppm) | 1.231 | 1.133,1.338 | <0.001 |
|  | SO_2_ (ppb) | 1.037 | 0.972,1.107 | 0.260 |
|  | NO (ppb) | 1.219 | 1.139,1.305 | <0.001 |
|  | NO_2_ (ppb) | 1.310 | 1.139,1.507 | <0.001 |
|  | NO_X_ (ppb) | 1.310 | 1.186,1.447 | <0.001 |
| 60-day | | | | |
|  | PM_10_ (µg/m^3^) | 0.952 | 0.843,1.075 | 0.432 |
|  | O_3_ (ppb) | 0.990 | 0.887,1.106 | 0.869 |
|  | CO (ppm) | 1.224 | 1.124,1.332 | <0.001 |
|  | SO_2_ (ppb) | 1.045 | 0.975,1.121 | 0.207 |
|  | NO (ppb) | 1.222 | 1.142,1.308 | <0.001 |
|  | NO_2_ (ppb) | 1.334 | 1.150,1.548 | <0.001 |
|  | NO_X_ (ppb) | 1.345 | 1.209,1.496 | <0.001 |
| 90-day | | | | |
|  | PM_10_ (µg/m^3^) | 0.943 | 0.831,1.069 | 0.360 |
|  | O_3_ (ppb) | 1.007 | 0.906,1.119 | 0.890 |
|  | CO (ppm) | 1.192 | 1.106,1.285 | <0.001 |
|  | SO_2_ (ppb) | 1.025 | 0.970,1.083 | 0.375 |
|  | NO (ppb) | 1.220 | 1.140,1.306 | <0.001 |
|  | NO_2_ (ppb) | 1.334 | 1.149,1.548 | <0.001 |
|  | NO_X_ (ppb) | 1.348 | 1.210,1.501 | <0.001 |
| 180-day | | | | |
|  | PM_10_ (µg/m^3^) | 0.960 | 0.852,1.080 | 0.499 |
|  | O_3_ (ppb) | 0.994 | 0.872,1.134 | 0.940 |
|  | CO (ppm) | 1.228 | 1.122,1.344 | <0.001 |
|  | SO_2_ (ppb) | 1.015 | 0.964,1.068 | 0.559 |
|  | NO (ppb) | 1.251 | 1.156,1.353 | <0.001 |
|  | NO_2_ (ppb) | 1.257 | 1.097,1.439 | 0.001 |
|  | NO_X_ (ppb) | 1.324 | 1.192,1.471 | <0.001 |
| 1-year | | | | |
|  | PM_10_ (µg/m^3^) | 0.992 | 0.901,1.093 | 0.884 |
|  | O_3_ (ppb) | 1.053 | 0.898,1.233 | 0.521 |
|  | CO (ppm) | 1.180 | 1.102,1.265 | <0.001 |
|  | SO_2_ (ppb) | 1.037 | 0.964,1.116 | 0.324 |
|  | NO (ppb) | 1.187 | 1.116,1.262 | <0.001 |
|  | NO_2_ (ppb) | 1.209 | 1.094,1.336 | <0.001 |
|  | NO_X_ (ppb) | 1.219 | 1.130,1.314 | <0.001 |

^a^ Model adjusted for sex, age, marital status, educational attainment, financial status (self-reported), physical activity, alcohol intake, heart disease, IADL score, season of study visit, and SPMSQ score. The ORs and 95% CIs for each increase of interquartile range (IQR) in PM_10_, O_3_, CO, SO_2_, NO, NO_2_, and NOx are expressed.

**Supplementary** **Table S7**. Two-pollutant model of air pollution exposure and its association with depressive symptoms among older adults.

| Exposure window | | CESD  <10 VS. ≥10 | | |
| --- | --- | --- | --- | --- |
|  |  | AOR ^e^ | 95%CI | p-value |
| 7-day | | | | |
|  | NO_X_ (ppb)^a^ | 1.303 | 1.150,1.477 | <0.001 |
|  | NO_X_ (ppb)^b^ | 1.383 | 1.214,1.576 | <0.001 |
|  | NO_X_ (ppb)^c^ | 1.335 | 1.174,1.520 | <0.001 |
|  | NO_X_ (ppb)^d^ | 1.202 | 0.922,1.568 | 0.172 |
| 14-day | | | | |
|  | NO_X_ (ppb)^a^ | 1.271 | 1.157,1.396 | <0.001 |
|  | NO_X_ (ppb)^b^ | 1.312 | 1.193,1.444 | <0.001 |
|  | NO_X_ (ppb)^c^ | 1.277 | 1.161,1.404 | <0.001 |
|  | NO_X_ (ppb)^d^ | 1.219 | 0.974,1.526 | 0.082 |
| 21-day | | | | |
|  | NO_X_ (ppb)^a^ | 1.297 | 1.178,1.429 | <0.001 |
|  | NO_X_ (ppb)^b^ | 1.338 | 1.212,1.476 | <0.001 |
|  | NO_X_ (ppb)^c^ | 1.292 | 1.174,1.423 | <0.001 |
|  | NO_X_ (ppb)^d^ | 1.247 | 0.977,1.593 | 0.075 |
| 30-day | | | | |
|  | NO_X_ (ppb)^a^ | 1.311 | 1.184,1.451 | <0.001 |
|  | NO_X_ (ppb)^b^ | 1.353 | 1.220,1.502 | <0.001 |
|  | NO_X_ (ppb)^c^ | 1.308 | 1.182,1.447 | <0.001 |
|  | NO_X_ (ppb)^d^ | 1.307 | 1.014,1.683 | 0.038 |
| 60-day | | | | |
|  | NO_X_ (ppb)^a^ | 1.348 | 1.209,1.502 | <0.001 |
|  | NO_X_ (ppb)^b^ | 1.397 | 1.248,1.565 | <0.001 |
|  | NO_X_ (ppb)^c^ | 1.342 | 1.205,1.496 | <0.001 |
|  | NO_X_ (ppb)^d^ | 1.479 | 1.141,1.916 | 0.003 |
| 90-day | | | | |
|  | NO_X_ (ppb)^a^ | 1.346 | 1.207,1.502 | <0.001 |
|  | NO_X_ (ppb)^b^ | 1.401 | 1.250,1.570 | <0.001 |
|  | NO_X_ (ppb)^c^ | 1.349 | 1.209,1.505 | <0.001 |
|  | NO_X_ (ppb)^d^ | 1.526 | 1.165,1.999 | 0.002 |
| 180-day | | | | |
|  | NO_X_ (ppb)^a^ | 1.323 | 1.190,1.470 | <0.001 |
|  | NO_X_ (ppb)^b^ | 1.393 | 1.243,1.562 | <0.001 |
|  | NO_X_ (ppb)^c^ | 1.333 | 1.197,1.485 | <0.001 |
|  | NO_X_ (ppb)^d^ | 1.541 | 1.159,2.047 | 0.002 |
| 1-year | | | | |
|  | NO_X_ (ppb)^a^ | 1.229 | 1.138,1.329 | <0.001 |
|  | NO_X_ (ppb)^b^ | 1.257 | 1.161,1.361 | <0.001 |
|  | NO_X_ (ppb)^c^ | 1.222 | 1.131,1.321 | <0.001 |
|  | NO_X_ (ppb)^d^ | 1.284 | 1.025,1.608 | 0.029 |

^a^ Model with adjustments for sex, age, educational attainment, marital status, financial status (self-reported), physical activity, alcohol intake, heart disease, IADL score, season of study visit, SPMSQ score, and exposure to PM_10_.

^b^ Model with adjustments for sex, age, educational attainment, marital status, financial status (self-reported), physical activity, alcohol intake, heart disease, IADL score, season of study visit, SPMSQ score, and exposure to O_3_.

^c^ Model with adjustments for sex, age, educational attainment, marital status, financial status (self-reported), physical activity, alcohol intake, heart disease, IADL score, season of study visit, SPMSQ score, and exposure to SO_2_.

^d^ Model with adjustments for sex, age, educational attainment, marital status, financial status (self-reported), physical activity, alcohol intake, heart disease, IADL score, season of study visit, SPMSQ score, and exposure to CO.

^e^ The ORs and 95% CIs for each increase of interquartile range (IQR) in **NOx** is expressed.

**Supplementary** **Table S8**. Two-pollutant model of air pollution exposure and its association with depressive symptoms among older adults.

| Exposure window | | CESD  <10 VS. ≥10 | | |
| --- | --- | --- | --- | --- |
|  |  | AOR ^e^ | 95%CI | p-value |
| 7-day | | | | |
|  | CO (ppm)^a^ | 1.222 | 1.106,1.351 | <0.001 |
|  | CO (ppm)^b^ | 1.259 | 1.138,1.393 | <0.001 |
|  | CO (ppm)^c^ | 1.232 | 1.116,1.361 | <0.001 |
|  | CO (ppm)^d^ | 1.082 | 0.876,1.336 | 0.462 |
| 14-day | | | | |
|  | CO (ppm)^a^ | 1.235 | 1.131,1.347 | <0.001 |
|  | CO (ppm)^b^ | 1.252 | 1.148,1.365 | <0.001 |
|  | CO (ppm)^c^ | 1.234 | 1.133,1.345 | <0.001 |
|  | CO (ppm)^d^ | 1.046 | 0.849,1.288 | 0.671 |
| 21-day | | | | |
|  | CO (ppm)^a^ | 1.219 | 1.128,1.318 | <0.001 |
|  | CO (ppm)^b^ | 1.227 | 1.137,1.324 | <0.001 |
|  | CO (ppm)^c^ | 1.215 | 1.126,1.310 | <0.001 |
|  | CO (ppm)^d^ | 1.032 | 0.849,1.255 | 0.746 |
| 30-day | | | | |
|  | CO (ppm)^a^ | 1.233 | 1.132,1.342 | <0.001 |
|  | CO (ppm)^b^ | 1.241 | 1.141,1.349 | <0.001 |
|  | CO (ppm)^c^ | 1.230 | 1.132,1.337 | <0.001 |
|  | CO (ppm)^d^ | 1.002 | 0.811,1.238 | 0.983 |
| 60-day | | | | |
|  | CO (ppm)^a^ | 1.225 | 1.124,1.336 | <0.001 |
|  | CO (ppm)^b^ | 1.235 | 1.132,1.347 | <0.001 |
|  | CO (ppm)^c^ | 1.221 | 1.121,1.329 | <0.001 |
|  | CO (ppm)^d^ | 0.920 | 0.748,1.131 | 0.430 |
| 90-day | | | | |
|  | CO (ppm)^a^ | 1.192 | 1.104,1.287 | <0.001 |
|  | CO (ppm)^b^ | 1.200 | 1.112,1.296 | <0.001 |
|  | CO (ppm)^c^ | 1.193 | 1.106,1.286 | <0.001 |
|  | CO (ppm)^d^ | 0.910 | 0.754,1.097 | 0.325 |
| 180-day | | | | |
|  | CO (ppm)^a^ | 1.227 | 1.120,1.344 | <0.001 |
|  | CO (ppm)^b^ | 1.251 | 1.138,1.375 | <0.001 |
|  | CO (ppm)^c^ | 1.228 | 1.122,1.345 | <0.001 |
|  | CO (ppm)^d^ | 0.869 | 0.682,1.108 | 0.260 |
| 1-year | | | | |
|  | CO (ppm)^a^ | 1.194 | 1.111,1.283 | <0.001 |
|  | CO (ppm)^b^ | 1.205 | 1.122,1.295 | <0.001 |
|  | CO (ppm)^c^ | 1.180 | 1.101,1.264 | <0.001 |
|  | CO (ppm)^d^ | 0.950 | 0.773,1.168 | 0.629 |

^a^ Model with adjustments for sex, age, educational attainment, marital status, financial status (self-reported), physical activity, alcohol intake, heart disease, IADL score, season of study visit, SPMSQ score, and exposure to PM_10_.

^b^ Model with adjustments for sex, age, educational attainment, marital status, financial status (self-reported), physical activity, alcohol intake, heart disease, IADL score, season of study visit, SPMSQ score, and exposure to O_3_.

^c^ Model with adjustments for sex, age, educational attainment, marital status, financial status (self-reported), physical activity, alcohol intake, heart disease, IADL score, season of study visit, SPMSQ score, and exposure to SO_2_.

^d^ Model with adjustments for sex, age, educational attainment, marital status, financial status (self-reported), physical activity, alcohol intake, heart disease, IADL score, season of study visit, SPMSQ score, and exposure to NOx.

^e^ The ORs and 95% CIs for each increase of interquartile range (IQR) in **CO** is expressed.

**Supplementary** **Table S9**. Two-pollutant model of CO residual exposure and NOx with depressive symptoms among older adults.

| Exposure window | | CESD  <10 VS.≥10 | | |
| --- | --- | --- | --- | --- |
|  |  | AOR^c^ | 95%CI | p-value |
| 7-day | | | | |
|  | CO residual (ppm)^a^ | 1.211 | 0.522, 2.809 | 0.656 |
|  | NO_X_ (ppb)^b^ | 1.015 | 1.008, 1.022 | <0.001 |
| 14-day | | | | |
|  | CO residual (ppm)^a^ | 1.195 | 0.510, 2.804 | 0.681 |
|  | NO_X_ (ppb)^b^ | 1.016 | 1.010, 1.023 | <0.001 |
| 21-day | | | | |
|  | CO residual (ppm)^a^ | 1.103 | 0.497, 2.449 | 0.810 |
|  | NO_X_ (ppb)^b^ | 1.015 | 1.009, 1.021 | <0.001 |
| 30-day | | | | |
|  | CO residual (ppm)^a^ | 0.975 | 0.420, 2.261 | 0.953 |
|  | NO_X_ (ppb)^b^ | 1.016 | 1.010, 1.022 | <0.001 |
| 60-day | | | | |
|  | CO residual (ppm)^a^ | 0.779 | 0.330, 1.834 | 0.567 |
|  | NO_X_ (ppb)^b^ | 1.017 | 1.010, 1.023 | <0.001 |
| 90-day | | | | |
|  | CO residual (ppm)^a^ | 0.690 | 0.271, 1.759 | 0.437 |
|  | NO_X_ (ppb)^b^ | 1.017 | 1.010, 1.023 | <0.001 |
| 180-day | | | | |
|  | CO residual (ppm)^a^ | 0.641 | 0.186, 2.211 | 0.482 |
|  | NO_X_ (ppb)^b^ | 1.017 | 1.010, 1.023 | <0.001 |
| 1-year | | | | |
|  | CO residual(ppm)^a^ | 0.656 | 0.254, 1.693 | 0.383 |
|  | NO_X_(ppb)^b^ | 1.017 | 1.010, 1.023 | <0.001 |

^a^ Model with adjustments for sex, age, educational attainment, marital status, financial status (self-reported), physical activity, alcohol intake, heart disease, IADL score, season of study visit, SPMSQ score, and exposure to NOx.

^b^ Model with adjustments for sex, age, educational attainment, marital status, financial status (self-reported), physical activity, alcohol intake, heart disease, IADL score, season of study visit, SPMSQ score, and exposure to CO residual.

^c^ The ORs and 95% CIs for each increase of one unite in CO residual and NOx are expressed.

**Supplementary** **Table S10**. Generalized linear mixed models of the associations between air pollution exposure (Q1—Q4) and depressive symptoms in older adults (n = 5776)

| **Air pollution** | **Depressive symptoms ≥ 10** | |  | **P value** |
| --- | --- | --- | --- | --- |
|  | **AOR** | **95% (CI)** |  |  |
| **7-days** |  |  |  |  |
| **PM_10_(µg/m^3^)** |  |  |  |  |
| Q1 | Ref. |  |  |  |
| Q2 | 1.109 | (0.886, 1.389) |  | 0.365 |
| Q3 | 0.918 | (0.734, 1.149) |  | 0.456 |
| Q4 | 0.884 | (0.704, 1.110) |  | 0.288 |
| **O_3_(ppb)** |  |  |  |  |
| Q1 | Ref. |  |  |  |
| Q2 | 1.546 | (1.221,1.958) |  | 0.000 |
| Q3 | 1.509 | (1.187, 1.920) |  | 0.001 |
| Q4 | 1.524 | (1.199, 1.938) |  | 0.001 |
| **CO(ppm)** |  |  |  |  |
| Q1 | Ref. |  |  |  |
| Q2 | 1.000 | (0.795, 1.258) |  | 0.999 |
| Q3 | 0.980 | (0.778, 1.234) |  | 0.861 |
| Q4 | 1.215 | (0.970, 1.522) |  | 0.090 |
| **SO_2_(ppb)** |  |  |  |  |
| Q1 | Ref. |  |  |  |
| Q2 | 1.099 | (0.882,1.369) |  | 0.399 |
| Q3 | 0.998 | (0.799, 1.247) |  | 0.988 |
| Q4 | 0.981 | (0.791, 1.216) |  | 0.861 |
| **NO(ppb)** |  |  |  |  |
| Q1 | Ref. |  |  |  |
| Q2 | 1.010 | (0.805, 1.268) |  | 0.932 |
| Q3 | 1.073 | (0.868, 1.326) |  | 0.514 |
| Q4 | 1.412 | (1.132, 1.762) |  | 0.002 |
| **NO_2_(ppb)** |  |  |  |  |
| Q1 | Ref. |  |  |  |
| Q2 | 1.247 | (0.987, 1.577) |  | 0.064 |
| Q3 | 1.369 | (1.082, 1.733) |  | 0.009 |
| Q4 | 1.353 | (1.083, 1.690) |  | 0.008 |
| **NOx(ppb)** |  |  |  |  |
| Q1 | Ref. |  |  |  |
| Q2 | 1.353 | (1.074, 1.704) |  | 0.010 |
| Q3 | 1.095 | (0.873, 1.373) |  | 0.432 |
| Q4 | 1.524 | (1.215, 1.911) |  | <0.001 |

Model adjusted for sex, age, marital status, educational attainment, financial status (self-reported), physical activity, alcohol intake, heart disease, IADL score, season of study visit, and SPMSQ score.

**Supplementary** **Table S11**. Generalized linear mixed models of the associations between air pollution exposure (Q1—Q4) and depressive symptoms in older adults (n = 5776)

| **Air pollution** | **Depressive symptoms ≥ 10** | |  | **P value** |
| --- | --- | --- | --- | --- |
|  | **AOR** | **95% (CI)** |  |  |
| **14-days** |  |  |  |  |
| **PM_10_(µg/m^3^)** |  |  |  |  |
| Q1 | Ref. |  |  |  |
| Q2 | 1.031 | (0.826, 1.287) |  | 0.790 |
| Q3 | 0.876 | (0.691, 1.109) |  | 0.270 |
| Q4 | 0.861 | (0.682, 1.086) |  | 0.207 |
| **O_3_(ppb)** |  |  |  |  |
| Q1 | Ref. |  |  |  |
| Q2 | 1.623 | (1.283, 2.053) |  | <0.001 |
| Q3 | 1.393 | (1.109, 1.750) |  | 0.004 |
| Q4 | 1.309 | (1.028, 1.666) |  | 0.029 |
| **CO(ppm)** |  |  |  |  |
| Q1 | Ref. |  |  |  |
| Q2 | 1.041 | (0.823, 1.317) |  | 0.737 |
| Q3 | 1.002 | (0.786, 1.278) |  | 0.987 |
| Q4 | 1.469 | (1.160, 1.862) |  | 0.001 |
| **SO_2_(ppb)** |  |  |  |  |
| Q1 | Ref. |  |  |  |
| Q2 | 1.065 | (0.859, 1.321) |  | 0.566 |
| Q3 | 1.283 | (1.008, 1.632) |  | 0.043 |
| Q4 | 1.223 | (0.985, 1.518) |  | 0.068 |
| **NO(ppb)** |  |  |  |  |
| Q1 | Ref. |  |  |  |
| Q2 | 1.003 | (0.795, 1.266) |  | 0.980 |
| Q3 | 1.189 | (0.941,1.502) |  | 0.147 |
| Q4 | 1.539 | (1.224, 1.934) |  | 0.000 |
| **NO_2_(ppb)** |  |  |  |  |
| Q1 | Ref. |  |  |  |
| Q2 | 1.481 | (1.171, 1.872) |  | 0.001 |
| Q3 | 1.205 | (0.948, 1.533) |  | 0.128 |
| Q4 | 1.599 | (1.277, 2.003) |  | <0.001 |
| **NOx(ppb)** |  |  |  |  |
| Q1 | Ref. |  |  |  |
| Q2 | 1.403 | (1.112, 1.770) |  | 0.004 |
| Q3 | 1.267 | (1.010, 1.589) |  | 0.041 |
| Q4 | 1.755 | (1.387, 2.221) |  | <0.001 |

Model adjusted for sex, age, marital status, educational attainment, financial status (self-reported), physical activity, alcohol intake, heart disease, IADL score, season of study visit, and SPMSQ score.

**Supplementary** **Table S12**. Generalized linear mixed models of the associations between air pollution exposure (Q1—Q4) and depressive symptoms in older adults (n = 5776)

| **Air pollution** | **Depressive symptoms ≥ 10** | |  | **P value** |
| --- | --- | --- | --- | --- |
|  | **AOR** | **95% (CI)** |  |  |
| **21-days** |  |  |  |  |
| **PM_10_(µg/m^3^)** |  |  |  |  |
| Q1 | Ref. |  |  |  |
| Q2 | 1.111 | (0.886, 1.393) |  | 0.361 |
| Q3 | 0.874 | (0.698, 1.094) |  | 0.240 |
| Q4 | 0.915 | (0.727, 1.152) |  | 0.451 |
| **O_3_(ppb)** |  |  |  |  |
| Q1 | Ref. |  |  |  |
| Q2 | 1.367 | (1.084, 1.723) |  | 0.008 |
| Q3 | 1.686 | (1.335, 2.130) |  | 0.000 |
| Q4 | 1.180 | (0.918, 1.517) |  | 0.196 |
| **CO(ppm)** |  |  |  |  |
| Q1 | Ref. |  |  |  |
| Q2 | 0.961 | (0.756, 1.222) |  | 0.747 |
| Q3 | 0.911 | (0.723, 1.149) |  | 0.432 |
| Q4 | 1.365 | (1.084, 1.719) |  | 0.008 |
| **SO_2_(ppb)** |  |  |  |  |
| Q1 | Ref. |  |  |  |
| Q2 | 1.138 | (0.899, 1.440) |  | 0.283 |
| Q3 | 1.326 | (1.040, 1.690) |  | 0.023 |
| Q4 | 1.286 | (1.017, 1.627) |  | 0.036 |
| **NO(ppb)** |  |  |  |  |
| Q1 | Ref. |  |  |  |
| Q2 | 1.193 | (0.944, 1.509) |  | 0.139 |
| Q3 | 1.108 | (0.882, 1.392) |  | 0.380 |
| Q4 | 1.662 | (1.327, 2.082) |  | <0.001 |
| **NO_2_(ppb)** |  |  |  |  |
| Q1 | Ref. |  |  |  |
| Q2 | 1.443 | (1.145, 1.818) |  | 0.002 |
| Q3 | 1.250 | (0.984, 1.588) |  | 0.067 |
| Q4 | 1.597 | (1.271, 2.007) |  | <0.001 |
| **NOx(ppb)** |  |  |  |  |
| Q1 | Ref. |  |  |  |
| Q2 | 1.396 | (1.105, 1.763) |  | 0.005 |
| Q3 | 1.235 | (0.986, 1.547) |  | 0.066 |
| Q4 | 1.825 | (1.444, 2.308) |  | <0.001 |

Model adjusted for sex, age, marital status, educational attainment, financial status (self-reported), physical activity, alcohol intake, heart disease, IADL score, season of study visit, and SPMSQ score.

**Supplementary** **Table S13**. Generalized linear mixed models of the associations between air pollution exposure (Q1—Q4) and depressive symptoms in older adults (n = 5776)

| **Air pollution** | **Depressive symptoms ≥ 10** | |  | **P value** |
| --- | --- | --- | --- | --- |
|  | **AOR** | **95% (CI)** |  |  |
| **30-days** |  |  |  |  |
| **PM_10_(µg/m^3^)** |  |  |  |  |
| Q1 | Ref. |  |  |  |
| Q2 | 1.109 | (0.889, 1.382) |  | 0.359 |
| Q3 | 0.975 | (0.773, 1.230) |  | 0.832 |
| Q4 | 0.905 | (0.722, 1.134) |  | 0.388 |
| **O_3_(ppb)** |  |  |  |  |
| Q1 | Ref. |  |  |  |
| Q2 | 1.518 | (1.216, 1.895) |  | <0.001 |
| Q3 | 1.545 | (1.239, 1.927) |  | <0.001 |
| Q4 | 1.157 | (0.898, 1.491) |  | 0.259 |
| **CO(ppm)** |  |  |  |  |
| Q1 | Ref. |  |  |  |
| Q2 | 0.875 | (0.688, 1.112) |  | 0.275 |
| Q3 | 0.857 | (0.684, 1.073) |  | 0.178 |
| Q4 | 1.315 | (1.049, 1.647) |  | 0.018 |
| **SO_2_(ppb)** |  |  |  |  |
| Q1 | Ref. |  |  |  |
| Q2 | 1.098 | (0.864, 1.395) |  | 0.446 |
| Q3 | 1.375 | (1.075, 1.759) |  | 0.011 |
| Q4 | 1.280 | (1.013, 1.618) |  | 0.038 |
| **NO(ppb)** |  |  |  |  |
| Q1 | Ref. |  |  |  |
| Q2 | 1.315 | (1.036, 1.669) |  | 0.024 |
| Q3 | 1.097 | (0.875, 1.374) |  | 0.423 |
| Q4 | 1.692 | (1.350, 1.212) |  | 0.000 |
| **NO_2_(ppb)** |  |  |  |  |
| Q1 | Ref. |  |  |  |
| Q2 | 1.453 | (1.148, 1.838) |  | 0.002 |
| Q3 | 1.335 | (1.038, 1.716) |  | 0.024 |
| Q4 | 1.657 | (1.295, 2.121) |  | <0.001 |
| **NOx(ppb)** |  |  |  |  |
| Q1 | Ref. |  |  |  |
| Q2 | 1.369 | (1.087, 1.724) |  | 0.008 |
| Q3 | 1.257 | (1.004, 1.574) |  | 0.046 |
| Q4 | 1.537 | (1.218, 1.939) |  | <0.001 |

Model adjusted for sex, age, marital status, educational attainment, financial status (self-reported), physical activity, alcohol intake, heart disease, IADL score, season of study visit, and SPMSQ score.

**Supplementary** **Table S14**. Generalized linear mixed models of the associations between air pollution exposure (Q1—Q4) and depressive symptoms in older adults (n = 5776)

| **Air pollution** | **Depressive symptoms ≥ 10** | |  | **P value** |
| --- | --- | --- | --- | --- |
|  | **AOR** | **95% (CI)** |  |  |
| **60-days** |  |  |  |  |
| **PM_10_(µg/m^3^)** |  |  |  |  |
| Q1 | Ref. |  |  |  |
| Q2 | 1.188 | (0.949, 1.486) |  | 0.132 |
| Q3 | 0.943 | (0.747, 1.191) |  | 0.623 |
| Q4 | 0.914 | (0.725, 1.153) |  | 0.449 |
| **O_3_(ppb)** |  |  |  |  |
| Q1 | Ref. |  |  |  |
| Q2 | 1.461 | (1.186, 1.801) |  | <0.001 |
| Q3 | 1.404 | (1.146, 1.719) |  | 0.001 |
| Q4 | 1.169 | (0.926, 1.476) |  | 0.189 |
| **CO(ppm)** |  |  |  |  |
| Q1 | Ref. |  |  |  |
| Q2 | 1.124 | (0.895, 1.412) |  | 0.314 |
| Q3 | 0.872 | (0.689, 1.103) |  | 0.254 |
| Q4 | 1.470 | (1.172, 1.845) |  | 0.001 |
| **SO_2_(ppb)** |  |  |  |  |
| Q1 | Ref. |  |  |  |
| Q2 | 1.155 | (0.873, 1.529) |  | 0.313 |
| Q3 | 0.990 | (0.791, 1.240) |  | 0.933 |
| Q4 | 1.178 | (0.953, 1.454) |  | 0.129 |
| **NO(ppb)** |  |  |  |  |
| Q1 | Ref. |  |  |  |
| Q2 | 1.161 | (0.920, 1465) |  | 0.209 |
| Q3 | 1.052 | (0.824, 1.343) |  | 0.684 |
| Q4 | 1.691 | (1.347, 2.122) |  | <0.001 |
| **NO_2_(ppb)** |  |  |  |  |
| Q1 | Ref. |  |  |  |
| Q2 | 1.495 | (1.183, 1.890) |  | 0.001 |
| Q3 | 1.416 | (1.097, 1.829) |  | 0.008 |
| Q4 | 1.704 | (1.326, 2.190) |  | <0.001 |
| **NOx(ppb)** |  |  |  |  |
| Q1 | Ref. |  |  |  |
| Q2 | 1.224 | (0.919, 1.632) |  | 0.167 |
| Q3 | 1.053 | (0.863, 1.284) |  | 0.611 |
| Q4 | 1.669 | (1.342, 2077) |  | <0.001 |

Model adjusted for sex, age, marital status, educational attainment, financial status (self-reported), physical activity, alcohol intake, heart disease, IADL score, season of study visit, and SPMSQ score.

**Supplementary** **Table S15**. Generalized linear mixed models of the associations between air pollution exposure (Q1—Q4) and depressive symptoms in older adults (n = 5776)

| **Air pollution** | **Depressive symptoms ≥ 10** | |  | **P value** |
| --- | --- | --- | --- | --- |
|  | **AOR** | **95% (CI)** |  |  |
| **90-days** |  |  |  |  |
| **PM_10_(µg/m^3^)** |  |  |  |  |
| Q1 | Ref. |  |  |  |
| Q2 | 1.541 | (1.206, 1.970) |  | 0.001 |
| Q3 | 1.091 | (0.835, 1.425) |  | 0.523 |
| Q4 | 1.141 | (0.875, 1.488) |  | 0.328 |
| **O_3_(ppb)** |  |  |  |  |
| Q1 | Ref. |  |  |  |
| Q2 | 1.567 | (1.279, 1919) |  | <0.001 |
| Q3 | 1.332 | (1.087, 1.633) |  | 0.006 |
| Q4 | 1.138 | (0.908, 1.425) |  | 0.262 |
| **CO(ppm)** |  |  |  |  |
| Q1 | Ref. |  |  |  |
| Q2 | 0.966 | (0.766, 1.217) |  | 0.767 |
| Q3 | 0.835 | (0.658, 1.059) |  | 0.138 |
| Q4 | 1.340 | (1.057, 1.699) |  | 0.016 |
| **SO_2_(ppb)** |  |  |  |  |
| Q1 | Ref. |  |  |  |
| Q2 | 1.372 | (1.063, 1.770) |  | 0.015 |
| Q3 | 1.353 | (1.050, 1.744) |  | 0.019 |
| Q4 | 1.190 | (0.918, 1.543) |  | 0.189 |
| **NO(ppb)** |  |  |  |  |
| Q1 | Ref. |  |  |  |
| Q2 | 1.047 | (0.837, 1.311) |  | 0.687 |
| Q3 | 0.953 | (0.751, 1.209) |  | 0.690 |
| Q4 | 1.598 | (1.279, 1.997) |  | <0.001 |
| **NO_2_(ppb)** |  |  |  |  |
| Q1 | Ref. |  |  |  |
| Q2 | 1.451 | (1.145, 1.839) |  | 0.002 |
| Q3 | 1.454 | (1.128, 1.875) |  | 0.004 |
| Q4 | 1.734 | (1.341, 2.243) |  | <0.001 |
| **NOx(ppb)** |  |  |  |  |
| Q1 | Ref. |  |  |  |
| Q2 | 1.580 | (1.247, 2.001) |  | <0.001 |
| Q3 | 1.297 | (1.014, 1.660) |  | 0.039 |
| Q4 | 1.681 | (1.303, 2.169) |  | <0.001 |

Model adjusted for sex, age, marital status, educational attainment, financial status (self-reported), physical activity, alcohol intake, heart disease, IADL score, season of study visit, and SPMSQ score.

**Supplementary** **Table S16**. Generalized linear mixed models of the associations between air pollution exposure (Q1—Q4) and depressive symptoms in older adults (n = 5776)

| **Air pollution** | **Depressive symptoms ≥ 10** | |  | **P value** |
| --- | --- | --- | --- | --- |
|  | **AOR** | **95% (CI)** |  |  |
| **180-days** |  |  |  |  |
| **PM_10_(µg/m^3^)** |  |  |  |  |
| Q1 | Ref. |  |  |  |
| Q2 | 1.043 | (0.840, 1.295) |  | 0.702 |
| Q3 | 0.870 | (0.686, 1.102) |  | 0.247 |
| Q4 | 0.844 | (0.672, 1.059) |  | 0.144 |
| **O_3_(ppb)** |  |  |  |  |
| Q1 | Ref. |  |  |  |
| Q2 | 1.491 | (1.214, 1.831) |  | <0.001 |
| Q3 | 1.402 | (1.123, 1.751) |  | 0.003 |
| Q4 | 1.101 | (0.863, 1.406) |  | 0.438 |
| **CO(ppm)** |  |  |  |  |
| Q1 | Ref. |  |  |  |
| Q2 | 0.800 | (0.633, 1.011) |  | 0.062 |
| Q3 | 0.816 | (0.651, 1.022) |  | 0.076 |
| Q4 | 1.324 | (1.071, 1.637) |  | 0.010 |
| **SO_2_(ppb)** |  |  |  |  |
| Q1 | Ref. |  |  |  |
| Q2 | 0.785 | (0.622, 0.989) |  | 0.040 |
| Q3 | 1.189 | (0.943, 1.498) |  | 0.143 |
| Q4 | 0.997 | (0.792, 1.255) |  | 0.979 |
| **NO(ppb)** |  |  |  |  |
| Q1 | Ref. |  |  |  |
| Q2 | 1.091 | (0.867, 1.372) |  | 0.459 |
| Q3 | 1.103 | (0.868, 1.403) |  | 0.421 |
| Q4 | 1.469 | (1.177, 1.833) |  | 0.001 |
| **NO_2_(ppb)** |  |  |  |  |
| Q1 | Ref. |  |  |  |
| Q2 | 1.387 | (1.102, 1.745) |  | 0.005 |
| Q3 | 1.381 | (1.064, 1.793) |  | 0.015 |
| Q4 | 1.649 | (1.290, 2.109) |  | 0.000 |
| **NOx(ppb)** |  |  |  |  |
| Q1 | Ref. |  |  |  |
| Q2 | 1.614 | (1.259, 2.070) |  | <0.001 |
| Q3 | 1.318 | (1.040, 1.672) |  | 0.022 |
| Q4 | 1.840 | (1.425, 2.376) |  | <0.001 |

Model adjusted for sex, age, marital status, educational attainment, financial status (self-reported), physical activity, alcohol intake, heart disease, IADL score, season of study visit, and SPMSQ score.

**Supplementary** **Table S17**. Generalized linear mixed models of the associations between air pollution exposure (Q1—Q4) and depressive symptoms in older adults (n = 5776)

| **Air pollution** | **Depressive symptoms ≥ 10** | |  | **P value** |
| --- | --- | --- | --- | --- |
|  | **AOR** | **95% (CI)** |  |  |
| **1-year** |  |  |  |  |
| **PM_10_(µg/m^3^)** |  |  |  |  |
| Q1 | Ref. |  |  |  |
| Q2 | 1.016 | (0.814, 1.266) |  | 0.891 |
| Q3 | 0.940 | (0.755, 1.170) |  | 0.577 |
| Q4 | 1.025 | (0.825, 1.273) |  | 0.826 |
| **O_3_(ppb)** |  |  |  |  |
| Q1 | Ref. |  |  |  |
| Q2 | 1.081 | (0.888, 1.317) |  | 0.437 |
| Q3 | 1.046 | (0.851, 1.288) |  | 0.667 |
| Q4 | 0.982 | (0.738, 1.305) |  | 0.898 |
| **CO(ppm)** |  |  |  |  |
| Q1 | Ref. |  |  |  |
| Q2 | 0.978 | (0.773, 1.237) |  | 0.852 |
| Q3 | 0.917 | (0.747, 1.125) |  | 0.404 |
| Q4 | 1.348 | (1.096, 1.658) |  | 0.005 |
| **SO_2_(ppb)** |  |  |  |  |
| Q1 | Ref. |  |  |  |
| Q2 | 1.219 | (0.970, 1.532) |  | 0.089 |
| Q3 | 1.302 | (1.024, 1.655) |  | 0.031 |
| Q4 | 1.363 | (1.091, 1.702) |  | 0.006 |
| **NO(ppb)** |  |  |  |  |
| Q1 | Ref. |  |  |  |
| Q2 | 0.835 | (0.678, 1.029) |  | 0.090 |
| Q3 | 1.086 | (0.868, 1.360) |  | 0.471 |
| Q4 | 1.367 | (1.113, 1.680) |  | 0.003 |
| **NO_2_(ppb)** |  |  |  |  |
| Q1 | Ref. |  |  |  |
| Q2 | 1.014 | (0.813, 1.265) |  | 0.902 |
| Q3 | 1.148 | (0.922, 1.430) |  | 0.218 |
| Q4 | 1.319 | (1.071, 1.624) |  | 0.009 |
| **NOx(ppb)** |  |  |  |  |
| Q1 | Ref. |  |  |  |
| Q2 | 1.007 | (0.809, 1.254) |  | 0.950 |
| Q3 | 1.022 | (0.823, 1.269) |  | 0.846 |
| Q4 | 1.435 | (1.161, 1.773) |  | 0.001 |

Model adjusted for sex, age, marital status, educational attainment, financial status (self-reported), physical activity, alcohol intake, heart disease, IADL score, season of study visit, and SPMSQ score.
